# Phosphorylation of TGIF2 represents a therapeutic target that drives EMT and metastasis of lung adenocarcinoma

**DOI:** 10.1186/s12885-023-10535-9

**Published:** 2023-01-16

**Authors:** Renle Du, Chen Wang, Jingjing Liu, Keyan Wang, Liping Dai, Wenzhi Shen

**Affiliations:** 1 Henan Institute of Medical and Pharmaceutical Sciences, Zhengzhou University, Zhengzhou, 450052 China; 2 College of Public Health, Zhengzhou University, Zhengzhou, 450052 Henan China; 3 State Key Laboratory of Esophageal Cancer Prevention & Treatment, Zhengzhou University, Zhengzhou, 450052 Henan China; 4 Henan Key Medical Laboratory of Tumor Molecular Biomarkers, Zhengzhou University, Zhengzhou, 450052 Henan China; 5 School of Basic Medical Sciences, Zhengzhou University, Zhengzhou, 450052 China; 6 Department of Pathology and Institute of Precision Medicine, Jining Medical University, Jining, 272067 China

**Keywords:** Lung adenocarcinoma, p-TGIF2, EMT, HDAC1, E-cadherin

## Abstract

**Background:**

TGF-β-induced factor homeobox 2 (TGIF2) is a transcription regulator that is phosphorylated by EGFR/ERK signaling. However, the functions of phosphorylated (p)-TGIF2 in cancer are largely unknown. Here, we investigated the roles of p-TGIF2 in promoting epithelial–mesenchymal transition (EMT) and metastasis in lung adenocarcinoma (LUAD).

**Methods:**

In vitro and in vivo experiments were conducted to investigate the role of TGIF2 in LUAD EMT and metastasis. Dual-luciferase reporter and ChIP assays were employed to observe the direct transcriptional regulation of E-cadherin by TGIF2 and HDAC1. Co-immunoprecipitation was performed to identify the interaction between TGIF2 and HDAC1.

**Results:**

Downregulating the expression of TGIF2 inhibited LUAD cell migration, EMT and metastasis in vitro and in vivo. Phosphorylation of TGIF2 by EGFR/ERK signaling was required for TGIF2-promoted LUAD EMT and metastasis since phosphorylation-deficient TGIF2 mutant lost these functions. Phosphorylation of TGIF2 was necessary to recruit HDAC1 to the E-cadherin promoter sequence and subsequently suppress E-cadherin transcription. Meanwhile, inhibition of HDAC1 repressed the TGIF2 phosphorylation-induced migration and EMT of LUAD cells. In xenograft mouse models, both inhibition of ERK and HDAC1 could significantly inhibited TGIF2-enhanced metastasis. Furthermore, TGIF2-positive staining was significantly correlated with E-cadherin-negative staining in human lung cancer specimens.

**Conclusions:**

Our study reveals the novel function of p-TGIF2 in promoting EMT and metastasis in LUAD; p-TGIF2 could be a potential therapeutic target to inhibit LUAD metastasis.

**Supplementary Information:**

The online version contains supplementary material available at 10.1186/s12885-023-10535-9.

## Background

Lung cancer is the leading cause of cancer-related mortality worldwide with a 5-year survival rate of less than 15% [1, 2]. Lung adenocarcinoma (LUAD) accounts for ~ 40% of all lung cancer cases and is the most frequently diagnosed subtype [3]. Despite improvements in our understanding of the pathogenesis of LUAD and therapeutic advances [4], tumor metastasis still represents a serious threat to patient survival.

Epithelial–mesenchymal transition (EMT) plays a key role in tumor development from initiation to metastasis [5, 6]. EMT is also associated with multiple other molecular processes, including tumor immune evasion [7]. During EMT, cancer cells lose their cell-to-cell contact by inhibiting epithelial (E)-cadherin expression and upregulating genes associated with the mesenchymal-cell phenotype, including those encoding N‑cadherin, fibronectin, and vimentin [8]. Consequently, loss of E‑cadherin expression has been associated with metastasis and poor prognosis [9]. Continuous EGF treatment has been shown to downregulate E-cadherin expression and result in the loss of cell–cell adherence junctions [10].

TGF-β-induced factor homeobox 2 (TGIF2) belongs to the TALE superfamily of homeodomain proteins that play important roles in many biological programs, including cell proliferation, embryonic development and differentiation. TGIF2 is a context-independent transcriptional regulator. The most extensively studied ability of TGIF2 is the recruitment of HDACs to DNA-bound Smad transcription complexes to induce the suppression of TGF-β signaling [11]. TGIF2 functions in the progression of several cancer types including ovarian, gastric, prostate and colorectal cancers [12–14]. TGIF2 was recently reported to promote cervical cancer metastasis by negatively regulating FCMR [15]. Being a target of microRNAs, TGIF2 is involved in various cancer-related processes [16–18]. We previously found that TGIF2 phosphorylation induced by EGFR/ERK signaling promoted LUAD stemness since phosphorylated (p)-TGIF2 showed increased stability [19]. However, the role of p-TGIF2 in LUAD metastasis remains largely unexplored.

In this study, we investigated the roles of TGIF2 in the EMT and metastasis of LUAD cells. We found that TGIF2 promoted EMT and metastasis by recruiting HDAC1 to downregulate E-cadherin expression. As a downstream target of EGF/EGFR/ERK signaling, TGIF2 phosphorylation was necessary for recruiting HDAC1 to the EMT program. Dual inhibition of ERK and HDAC1 significantly inhibited TGIF2 enhanced LUAD metastasis. The identification of p-TGIF2 as a key regulator of EMT and metastasis provides a promising target for LUAD therapy.

## Methods

### Cell culture

Human lung cancer cell lines A549 and H1299 were purchased from the American Type Culture Collection (ATCC, Washington, USA) and cultured in RIPM-1640 medium containing 10% fetal bovine serum (FBS), 0.1 mg/mL streptomycin and 100 U/mL penicillin. Cells were maintained at an atmosphere of 5% CO_2_% and 95% air at 37˚C. All cell lines were recently authenticated by cellular morphology and short tandem repeat profiling.

### Cell line establishment

Control or HDAC1-targeting shRNA templates were inserted into the pLV-H1-EF1a-Puro vector (Biosettia, San Diego, CA, USA) (named shCtrl, and shHDAC1). H1299 cells were infected with lentivirus expressing shHDAC1, followed by clone selection using 2 μg/mL puromycin to establish HDAC1 knockdown H1299 cells. TGIF2-silenced and TGIF2-rescue (shTGIF2#2 + TGIF2^resis^) stable H1299 and A549 cells, TGIF2^WT^-, TGIF2^DD^- and TGIF2^AA^-overexpressing H1299 cells were previously established [19]. The sequences of shRNA-HDAC1was: 5’-GGTGCTGTACATTGACATT-3’.

### Western blot

The western blot steps were described previously [20]. The blots were cut prior to hybridisation with antibodies during blotting. The original images of all blots are presented in the Supplementary Information. The proteins were detected using the following primary antibodies: anti-E-cadherin, HDAC1, Flag, vimentin and fibronectin (Cell Signaling Technology, Danvers, MA, USA). Anti-β-actin, TGIF2, ERK and p-ERK (Santa Cruz Biotechnology, Dallas, TX, USA).

### Real-time PCR

Real-time PCR was carried out according to the established protocols described previously [21]. The primer sequences are shown in Supplementary table S1.

### Immunohistochemistry (IHC) staining

The tissue microarrays were purchased from Alenabio Inc. (cat. #LC2081, Shanxi, China). The tissue sections were incubated with primary antibodies against TGIF2, E-cadherin, and Vimentin (1:200) overnight at 4 °C. The tissues were incubated with a peroxidase-conjugated secondary antibody at 37 °C for 1 h before the DAB Substrate Kit was used to reveal bound secondary antibody. The semiquantitative H score was used to estimate the immunoreactivity according to the established protocols described previously [22].

### Immunofluorescence staining

LUAD cell lines were fixed with 4% paraformaldehyde and were incubated with 5% goat serum for 1 h to block nonspecific interactions. Then cells were labeled with primary antibodies against E-cadherin, Vimentin, TGIF2, HDAC1 or Flag overnight at 4 °C, followed by incubation with fluorescent-dye-labeled secondary antibodies (1:200) (Thermo Fisher Scientific, Waltham, MA, USA) for 1 h. Nuclei were stained with DAPI. Finally, the images were captured with a confocal fluorescence microscope (Olympus, Tokyo, Japan).

### Dual-luciferase reporter assay

The *CDH1* gene promoter fragment was amplified from human genomic DNA and cloned into the firefly luciferase plasmid pGL3-basic-IRES. H1299 cells were transiently transfected with pGL3-*CDH1* reporter plasmid and TGIF2^WT^ or TGIF2^AA^ expression plasmids using Lipofectamine Reagent (Invitrogen, Waltham, MA, USA). After transfection for 36 h, luciferase activity was measured using the Dual-Luciferase Reporter Assay System (Promega, Madison, WI, USA). Luciferase activity was normalized to Renilla luciferase activity. The primer sequences used in this assay are listed in Supplementary table S2.

### Chromatin immunoprecipitation (ChIP)

ChIP was carried out using the EZ-Zyme Chromatin Prep Kit (Millipore, Billerica, MA, USA). Anti-TGIF2 antibody and Anti-HDAC1 antibody were used to precipitate DNA crosslinked with TGIF2 and HDAC1, anti-mouse IgG as a negative control. PCR and RT-PCR were performed to detect DNA fragments of the *CDH1* promoter region. Primers used in this assay are summarized in Supplementary tables S3,S4.

### Cell migration assays

For Transwell assays, 1 × 10^5^ cells were re-suspended in 200 μL of RPMI 1640 medium supplemented with 1% FBS and seeded into 5-μm pore Transwell chamber (Millipore, Burlington, MA, USA). Medium containing 10% FBS was added to the lower chamber. After incubation for 8 h, cells that migrating to the membrane surface were stained with 0.1% crystal violet staining solution. Four random fields were counted per chamber.

For wound healing assays, 1 × 10^6^ A549 or H1299 cells were seeded and grown to 90% confluence. A sterile pipette tip was used to create scratch wounds. Images were taken at 0 h, and 10 h (H1299) or 24 h (A549) with an IX71 inverted microscope (Olympus). Three independent experiments were performed.

### Immunoprecipitation

Wild type and Flag-TGIF2^AA^-overexpressing H1299 cell protein lysates were incubated with protein A/G agarose beads (Life Technologies) and antibody overnight at 4 °C. Then, the samples were centrifuged at 2500 rpm for 30 s. The pellets were washed with prechilled phosphate-buffered saline (PBS). Finally, bound proteins were boiled and analyzed by sodium dodecyl sulfate–polyacrylamide gel electrophoresis. Immunoprecipitates were analyzed by western blotting with anti-TGIF2 antibody (Santa Cruz Biotechnology, Dallas, TX, USA), anti-Flag antibody ((Sigma-Aldrich, St. Louis, MO, USA)) and anti-HDAC1 antibody (Cell Signaling Technology, Danvers, MA, USA).

### Animal studies

To confirm whether TGIF2 promotes metastasis in vivo. Six-week-old NOD/SCID male mice were purchased from SPF Biotechnology (Beijing, China). Mice were intravenously injected with 2 × 10^6^ TGIF2-silenced, TGIF2-rescued or control H1299 cells. Twenty days after tumor cell inoculation, the mice underwent bioluminescence imaging and were sacrificed. The lung tissues were fixed with formalin, embedded in paraffin, and subjected to HE staining or IHC staining. The percentage of lung metastatic foci was calculated using the formula: (area of metastatic foci/ total area of five lung lobes) × 100%. The area was measured using Photoshop software (Adobe, San Jose, CA, USA).

To determine whether p-TGIF2 was necessary for TGIF2-enhanced metastasis. 1.5 × 10^6^ H1299-vector, H1299-TGIF2^WT^ or H1299-TGIF2^AA^ cells were subcutaneously injected into each NOD/SCID mouse. Tumor volume was measured using calipers and calculated using the formula: (length × width^2^)/2. Thirty-five days after inoculation, the mice were sacrificed and subjected to lung metastatic analysis.

For ERK inhibitor and HDAC1 inhibitor treatments, 2 × 10^6^ TGIF2^WT^-overexpressing H1299 cells were injected into NOD/SCID mice via the tail vein. Twelve days after injection, mice were randomized into four groups (PBS, ERK inhibitor, HDAC1 inhibitor, ERK inhibitor + HDAC1 inhibitor). The mice were intraperitoneally injected with 50 mg/kg ERK inhibitor (SCH772984) (Selleck, Houston, Texas, USA) every day for 14 days. The mice received 80 mg/kg HDAC1 inhibitor (MGCD0103) (Selleck, Houston, Texas, USA) by daily intragastric administration for 14 days, and PBS was used as a control. Thirty days after inoculation, the metastatic foci in the lung were subjected to HE staining or IHC staining.

### Bioinformatics analysis

Gene expression profiles of 532 LUAD patients were downloaded from The Cancer Genome Atlas (TCGA) (https://cancergenome.nih.gov). Gene set enrichment analysis (GSEA) was performed according to the authors' guidelines published on the Broad Institute web pages (http://www.broadinstitute.org/gsea/index.jsp) with cell adhesion molecules signature and epithelial-mesenchymal transition gene set (from the MSigDB C2 curated gene sets collection) using Signal2Noise ratios. Survival analyses were conducted with the online tool (http://kmplot.com/analysis/index.php?p=service&cancer=lung) using the 2015 version. Patients with lung adenocarcinoma were selected for the First Progression (FP) and Post Progression Survival (PPS) assays. Log-rank was automatically computed.

### Statistical analysis

The data are presented as the mean ± standard deviation. A two-tailed Student’s *t*-test was performed to compare the differences between the two groups. A one-way ANOVA with post hoc Tukey’s Multiple Comparison Test was used to analyze the xenograft growth. A *p*-value of < 0.05 was used as the criterion for statistical significance. Spearman’s r test was used to determine the correlation between TGIF2 and E-cadherin. GraphPad Prism 8 was used to perform the statistical analyses.

## Results

### TGIF2 enhances LUAD cell migration and EMT in vitro

**Fig. 1:**
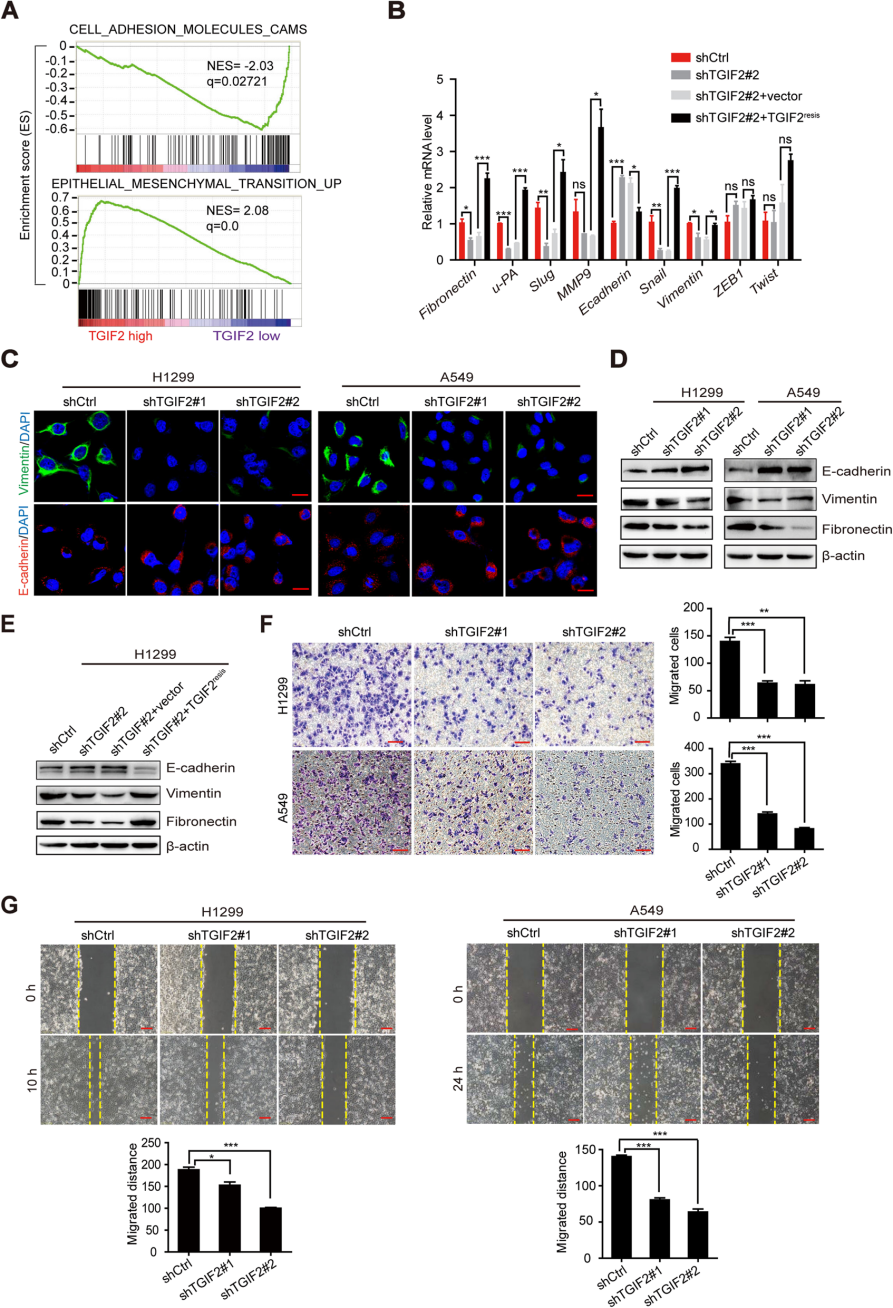
TGIF2 enhances LUAD cell migration and EMT in vitro. (**A**) GSEA of cell adhesion molecules enriched in TGIF2-low-expressing patients (upper panel). GSEA of EMT genes enriched in TGIF2-high-expressing patients (lower panel). (**B**) qRT-PCR analysis of EMT-related genes, including *Fibronectin*, *u-PA*, *Slug*, *MMP9*, *E-cadherin*, *Snail*, *Vimentin*, *ZEB1*, and *Twist*, in the indicated cells. (**C**) Immunofluorescence analysis of E-cadherin and Vimentin in A549 and H1299 cells with TGIF2 knockdown. Scale bars, 20 μm. (**D**) Western blot results of E-cadherin, Vimentin, and Fibronectin expression in TGIF2-knockdown A549 and H1299 cells. (**E**) Western blot results for E-cadherin, Vimentin, and Fibronectin expression in stable H1299 cells. (**F**) Transwell assay of the migration ability of TGIF2 knockdown A549 and H1299 cells. Scale bars, 100 μm. (**G**) Wound healing assay of the migrated distance of TGIF2 knockdown A549 and H1299 cells. Scale bars, 100 μm. *, *p* < 0.05; **, *p* < 0.01; ***, *p* < 0.001; ns, not significant. All data are representative of three repeated experiments

It was reported that cancer stem cell (CSC)-enriched subpopulation exhibits aspects of EMT activation [23, 24]. We previously found that TGIF2 can maintain CSC-like characteristics in LUAD [19]. In this study, gene set enrichment analysis (GSEA) revealed that a lower and higher expression of TGIF2 led to an enrichment of cell adhesion molecules and enrichment of EMT in LUAD patients, respectively (Fig. 1A), suggesting a role for TGIF2 in regulating EMT and metastasis in LUAD. In addition, we analyzed the correlation between the level of TGIF2 and the first progression (FP) and post progression survival (PPS) of patients with lung adenocarcinoma. We found that high TGIF2 expression was associated with poor clinical outcome in patients with LUAD (Fig. S1A and B).

To determine whether TGIF2 plays a role in EMT, several metastasis-related biomarkers were examined in TGIF2-silenced and shRNA-resistant TGIF2-overexpressing H1299 cells (Fig. S2A and B). We observed that *Fibronectin*, *U-Plasminogen Activator (U-PA)*, *Slug*, *Snail*, and *Vimentin* mRNA levels were positively associated with *TGIF2* expression, whereas the *E-cadherin* mRNA level was negatively associated with *TGIF2* expression (Fig. 1B). Silencing of TGIF2 increased the protein level of the key epithelial marker E-cadherin and decreased that of the mesenchymal marker Vimentin and Fibronectin (Fig. 1C and D), which was rescued by the ectopic expression of shRNA-resistant TGIF2 (TGIF2^resis^, Fig. 1E). We also observed that TGIF2-silenced A549 and H1299 cells showed a decreased capacity for migration (Fig. 1F and G), which was also rescued by ectopically expressed TGIF2 (Fig. S2C and S2D). These results highlight the ability of TGIF2 to promote LUAD cell migration and EMT.

### TGIF2 promotes EMT and metastasis of LUAD cells in vivo

**Fig. 2:**
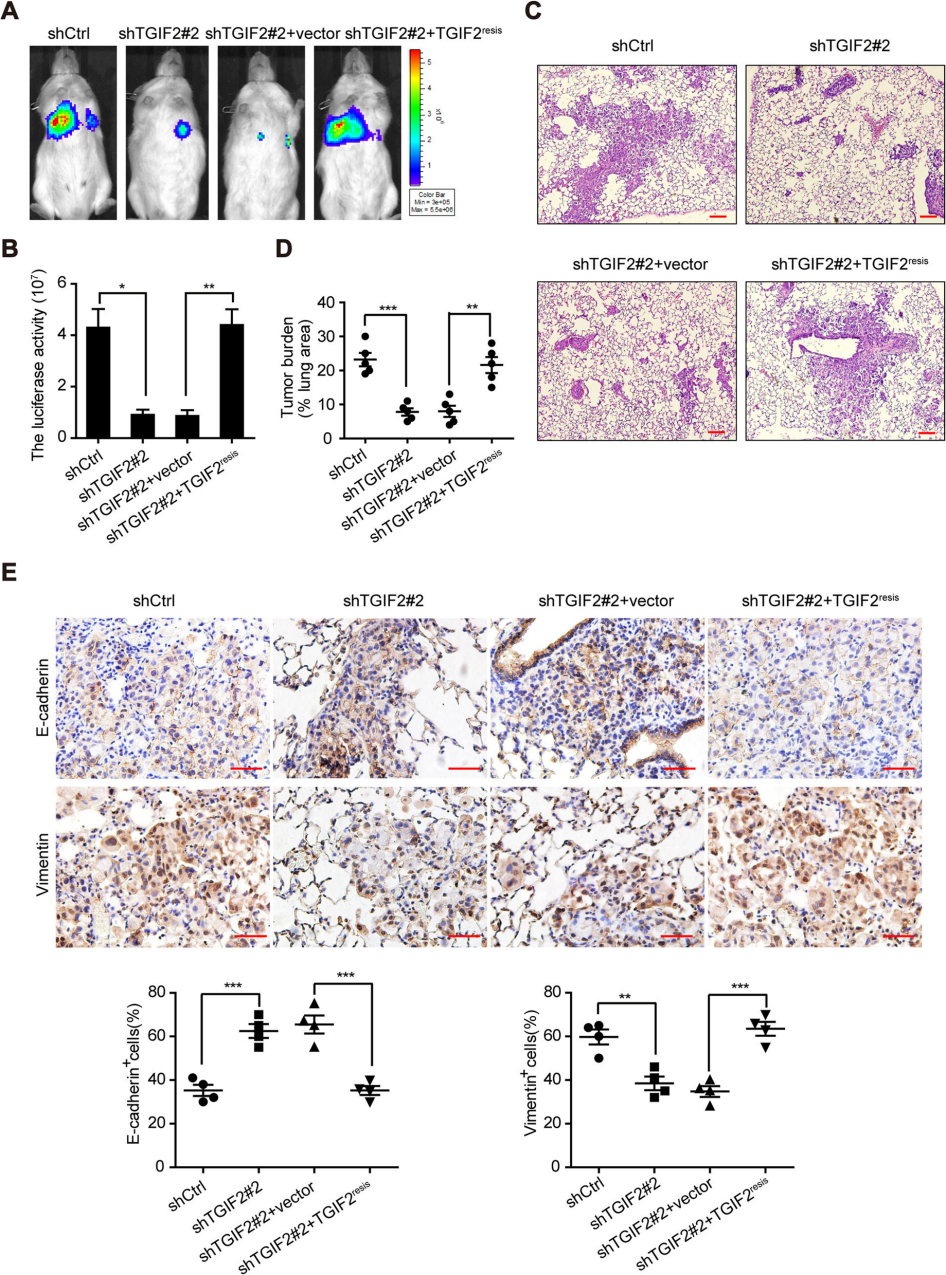
TGIF2 promotes EMT and metastasis of LUAD cells in vivo. (**A**) Bioluminescence imaging of pulmonary metastasis. Mice were intravenously injected with H1299-firefly luciferase stable cell strains and subjected to a bioluminescence test after 20 d. (**B**) Quantitation of bioluminescence signals in the indicated mice. Data are presented as the mean ± SEM (*n* = 6 mice per group). (**C**) H&E staining was used to analyze lung metastasis in the indicated mice. Scale bars, 250 μm. (**D**) Percentage of metastatic area in mouse lungs. (**E**) IHC analysis of E-cadherin and Vimentin in the indicated lung tumors (*n* = 4). Scale bars: 50 μm, upper panel. *, p < 0.05; **, *p* < 0.01; ***, *p* < 0.001. All data are representative of three repeated experiments

To examine the metastasis-promoting effects of TGIF2 in vivo, we established an intravenous mouse xenograft model by injecting H1299-luciferase cells. The silencing of TGIF2 significantly reduced metastasis according to in vivo bioluminescence imaging, which was rescued by the ectopic expression of TGIF2^resis^ (Fig. 2A, B). This result was further confirmed by counting the lung tumor burden (Fig. 2C, D). Furthermore, we observed increased levels of E-cadherin and decreased levels of vimentin in mouse lungs injected with TGIF2-knockdown cells, which was also be rescued by ectopically expressed TGIF2^resis^ (Fig. 2E). These results indicate that TGIF2 contributes to the EMT and metastasis of LUAD cells in vivo.

### Phosphorylation of TGIF2 by EGFR/ERK signaling is necessary for TGIF2-enhanced EMT and metastasis in LUAD

**Fig. 3:**
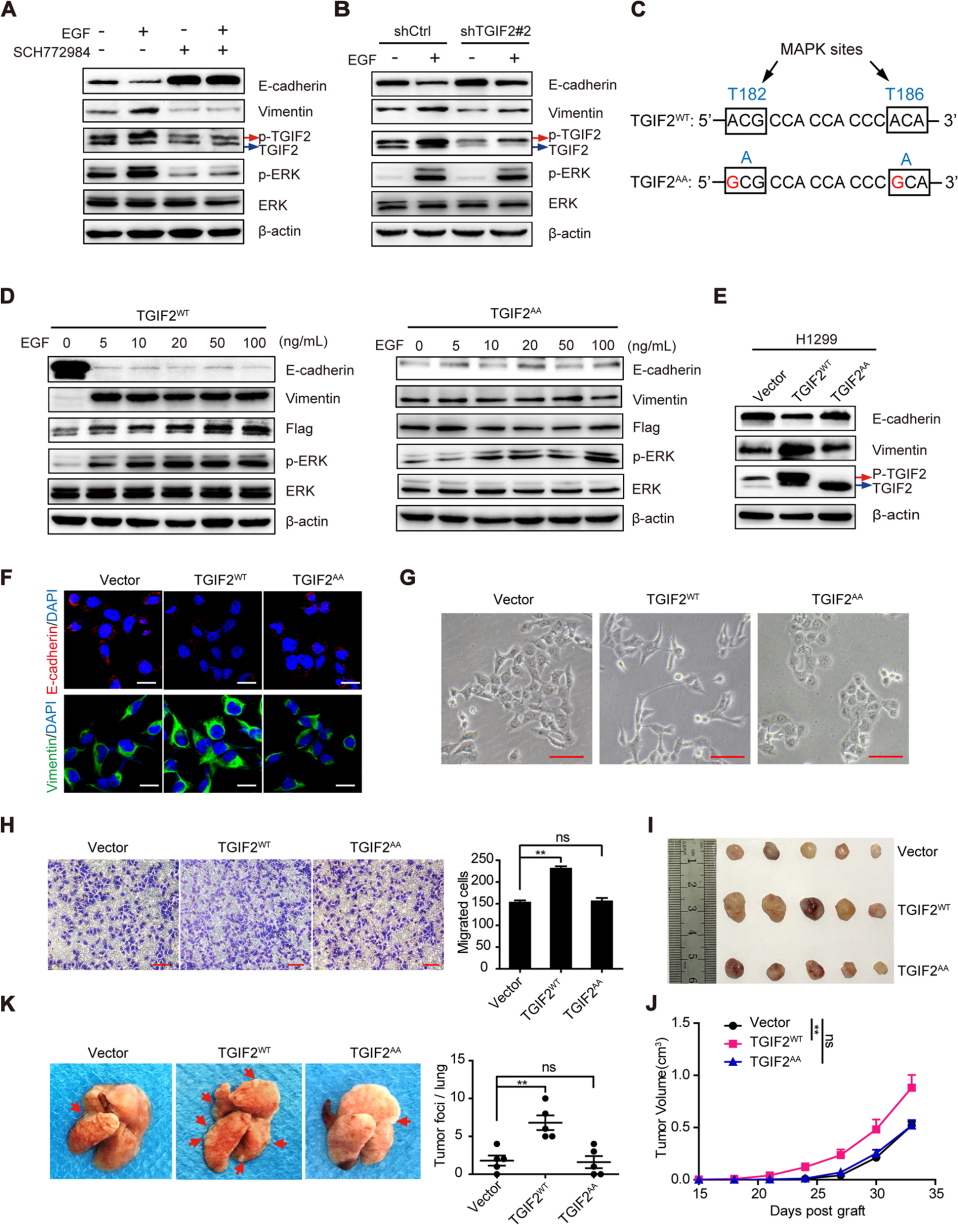
Phosphorylation of TGIF2 by EGFR/ERK signaling is necessary for TGIF2-enhanced EMT and metastasis in LUAD. (**A**) Western blot results of E-cadherin, Vimentin, TGIF2, ERK, and p-ERK expression in H1299 cells in the presence or absence of EGF and SCH772984. Cells were pre-incubated with 1 μM SCH772984 for 1 h and exposed to 50 ng/mL EGF for 5 min or 48 h. (**B**) Western blot results of E-cadherin, Vimentin, TGIF2, ERK, and p-ERK expression in TGIF2 knockdown and control H1299 cells in the presence or absence of EGF (50 ng/mL). (**C**) Schematic of the phosphorylation-deficient mutations (TGIF2^AA^) of the two MAPK sites in the TGIF2 coding sequence. (**D**) Western blots for Flag-tagged TGIF2^WT^ and TGIF2^AA^ in H1299 cells treated with the indicated dose of EGF for 5 min or 48 h. (**E**) Western blot results of TGIF2, p-TGIF2, E-cadherin, and Vimentin expression in H1299 cells overexpressing TGIF2^WT^ or TGIF2^AA^. (**F**) Immunofluorescence analysis of E-cadherin and Vimentin in H1299 cells with ectopic expression of TGIF2^WT^ or TGIF2^AA^. Scale bars, 20 μm. (**G**) Phase-contrast images showing the morphology of H1299 cells with ectopic expression of TGIF2^WT^ or TGIF2^AA^. Scale bars, 50 μm. (**H**) Transwell assay of H1299 cell migration according to the ectopic expression of TGIF2^WT^ or TGIF2^AA^. Scale bars, 100 μm. (**I**, **J**) Tumor presence (**I**) and growth curve (**J**) derived from different groups; 1.5 × 10^6^ H1299-vector, H1299-TGIF2^WT^, or H1299-TGIF2^AA^ cells were subcutaneously injected into each mouse. Tumors were excised and analyzed 35 d later. Data are presented as the mean ± SEM (*n* = 5 mice per group). (**K**) Representative lung images and number of metastatic foci in the different groups. **, *p* < 0.01; ns, not significant. All data shown are representative of three repeated experiments

We previously demonstrated that the phosphorylation of TGIF2 by EGF/EGFR/ERK signaling promoted the stemness of LUAD cells. To determine whether TGIF2 enhanced LUAD EMT in an EGFR/ERK signaling-dependent manner, H1299 cells were treated with EGF and ERK inhibitor (SCH772984, Fig. 3A). EGF decreased the expression of E-cadherin and increased the expression of vimentin, which was inhibited by ERK inhibition. Furthermore, we observed that the silencing of TGIF2 increased and decreased the EGF-induced expression of E-cadherin and vimentin, respectively (Fig. 3B).

To further investigate the role of TGIF2 downstream of EGFR/ERK signaling, we constructed phosphorylation-deficient TGIF2 mutants (i.e., T182A and T186A, or TGIF2^AA^ for short) that present a loss of phosphorylation at the MAP kinase sites (Fig. 3C). We observed that EGF could no longer induce EMT in TGIF2^AA^-transfected H1299 cells compared with that in TGIF2^WT^ cells (Fig. 3D). Next, we compared the functions of TGIF2 ^WT^ and TGIF2^AA^ in regulating EMT and metastasis. We found that the ectopic expression of TGIF2^WT^ reduced the expression of E-cadherin and enhanced the expression of vimentin, while TGIF2^AA^ had no effect (Fig. 3E, F). The ectopic expression of TGIF2^WT^ changed the cell morphology from epithelial to fibroblastic and spindle-shaped, in stark contrast to control cells, whereas the ectopic expression of TGIF2^AA^ induced a stellate-like phenotype in H1299 cells (Fig. 3G). Compared to the effect of TGIF2^WT^, TGIF2^AA^ did not increase the migration capacity of H1299 cells (Fig. 3H and S3). In the xenograft mouse model, overexpression of TGIF2^AA^ had no effect on tumor growth compared with that of TGIF2^WT^ (Fig. 3I, J). Consistent with the changes in vitro, TGIF2^WT^-transfected cells induced more metastatic nodules in the lungs compared with TGIF2^AA^-overexpressing or empty vector-expressing cells (Fig. 3K). Collectively, these results suggest that the phosphorylation of TGIF2 by EGFR/ERK signaling is necessary to promote EMT and metastasis in LUAD.

### p-TGIF2 downregulates the expression of E-cadherin by binding to its promoter

**Fig. 4:**
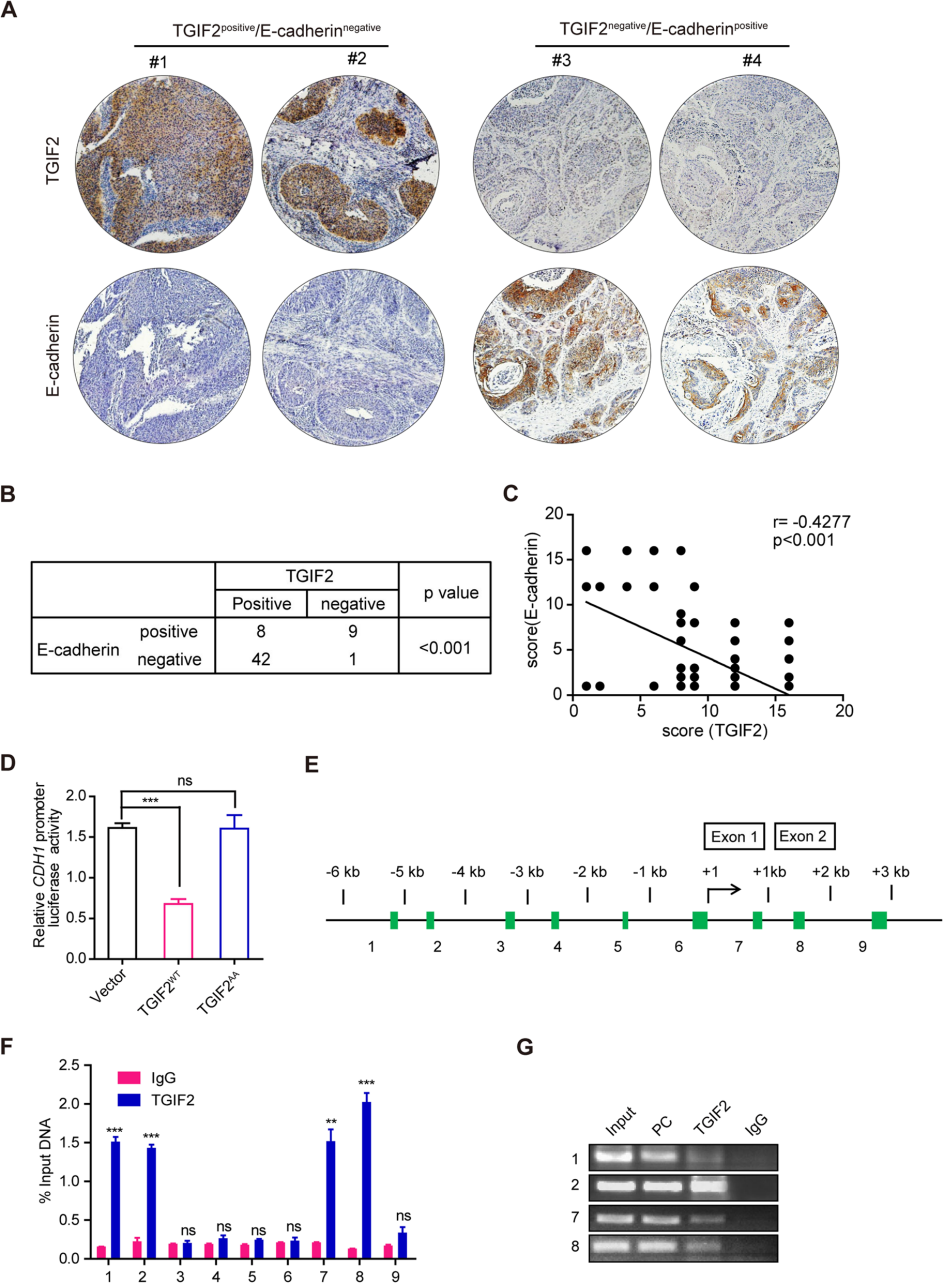
p-TGIF2 downregulates the expression of E-cadherin by binding to its promoter. (**A**) Representative IHC images of TGIF2 and E-cadherin expression in the NSCLC tissue array. (**B**) E-cadherin expression was assessed in 50 upregulated and 10 downregulated cases of TGIF2 expression. (**C**) Spearman’s correlation of the expression of E-cadherin and TGIF2 in human NSCLC tissues. (**D**) Luciferase assay of *CDH1* promoter activity. H1299 cells were transfected with pGL3-*CDH1*-promoter and empty vector, TGIF2^WT^, or TGIF2^AA^ and subjected to dual-luciferase analysis. (**E**) The promoter region of the *CDH1* gene scanned by nine amplicons. (**F**) TGIF2 ChIP-qPCR of the *CDH1* promoter at the indicated regions in H1299 cells. Mouse IgG served as a negative control. (**G**) ChIP assay with IgG and anti-TGIF2 antibody in H1299 cells followed by PCR. **, *p* < 0.01; ***, *p* < 0.001; ns, not significant. PC, positive control. All data are representative of three repeated experiments

In 60 human non-small cell lung cancer (NSCLC) samples, we found a negative correlation between the expression of TGIF2 and E-cadherin as determined by IHC staining (Fig. 4A, B, C). To explore whether phosphorylation of TGIF2 promoted LUAD EMT by regulating E-cadherin expression, we compared *CDH1* (codes for E-cadherin protein) promoter activity following transfection with TGIF2^WT^ or TGIF2^AA^. TGIF2^WT^ significantly decreased *CDH1* promoter activity, while TGIF2^AA^ had no effect (Fig. 4D). We performed a ChIP-qPCR assay to detect TGIF2 binding sites in the human *CDH1* gene from − 6 kb to + 3 kb in TGIF2^WT^-transfected H1299 cells (Fig. 4E). TGIF2 binding sites were found within the − 6 kb to − 4 kb and + 1 kb to + 2 kb regions of the *CDH1* promoter (Fig. 4F), which were further confirmed by a ChIP-PCR assay (Fig. 4G). These results demonstrate that the phosphorylation of TGIF2 is necessary for its binding to the *CDH1* promoter.

### p-TGIF2 recruits HDAC1 to promote LUAD EMT and metastasis

**Fig. 5:**
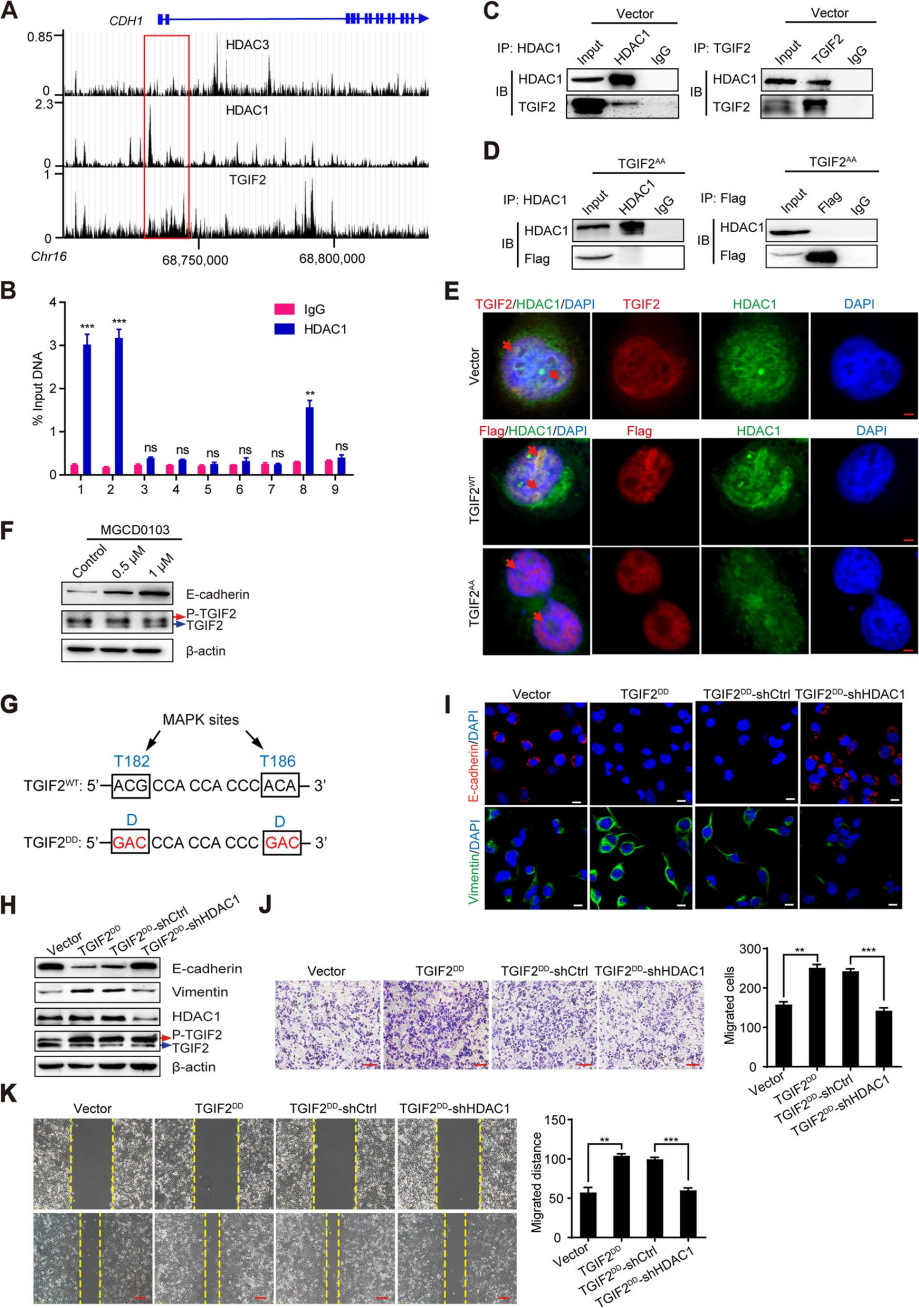
p-TGIF2 recruits HDAC1 to promote EMT and metastasis in LUAD. (**A**) ChIP-Seq data from the Cistrome Data Browser database showing the TGIF2, HDAC1 and HDAC3 binding sites on the human *CDH1* genomic locus. (**B**) ChIP-qPCR of HDAC1 on the *CDH1 *promoter at the indicated regions in H1299 cells. Mouse IgG served as a negative control. (**C**) Western blot assays of H1299 cell samples immunoprecipitated with anti-HDAC1 and anti-TGIF2 antibody. (**D**) Western blot assays of TGIF2^AA^-overexpressing H1299 cell samples immunoprecipitated with anti-HDAC1 and anti-Flag antibody. (**E**) Colocalization of TGIF2 and HDAC1 was examined in control and TGIF2^AA^-overexpressing H1299 cells by immunofluorescence. Scale bars, 5 μm. (**F**) Western blot for E-cadherin, TGIF2 and p-TGIF2 in H1299 cells with the indicated dose of HDAC1 inhibitor (MGCD0103). (**G**) Schematic of the phosphorylation-mimicking mutations (TGIF2^DD^) of the two MAPK sites in the TGIF2 coding sequence. (**H**) Western blots for E-cadherin, Vimentin, HDAC1, TGIF2 and p-TGIF2 in H1299 cells. (**I**) Immunofluorescence analysis of E-cadherin and Vimentin in the indicated H1299 cells. Scale bars, 20 μm. (**J**) Transwell assay of H1299 cell migration according to the ectopic expression of TGIF2^DD^ or HDAC1 knockdown. Scale bars, 100 μm. (**K**) Wound healing assay of the distance migrated by H1299 cells. Scale bars, 100 μm. **, *p* < 0.01; ***, *p* < 0.001; ns, not significant. All data are representative of three repeated experiments

We explored why the phosphorylation of TGIF2 was required for its binding to the *CDH1* promoter. Based on previous findings [11], we examined the involvement of HDACs in the regulation of E-cadherin expression by TGIF2. We searched the Cistrome Data Browser database and found that HDAC1, but not HDAC3, might bind to the − 6 kb to − 4 kb region of the *CDH1* promoter, where the TGIF2 binding sites were also shown (Fig. 5A). A ChIP-qPCR assay was performed and showed that HDAC1 bind to the region from − 6 kb to − 4 kb of the *CDH1* promoter (Fig. 5B), suggesting that TGIF2 might cooperate with HDAC1 to regulate *CDH1* transcription. To further confirm this result, we performed co-immunoprecipitation assays in empty vector- or TGIF2^AA^- transfected cells (Fig. 5C, D). Endogenous binding of p-TGIF2 with HDAC1 was observed in control cells, but not in phosphorylation-deficient TGIF2 mutants, which was confirmed by immunofluorescence analysis (Fig. 5E). We also observed increased E-cadherin expression after treatment with HDAC1 inhibitor (MGCD0103), but no effect was observed on TGIF2 expression (Fig. 5F). These results indicate that the phosphorylation of TGIF2 is required for HDAC1 recruitment to downregulate E-cadherin expression.

To determine the role of HDAC1 in p-TGIF2-induced EMT and metastasis, we generated a p-TGIF2 mimicking mutant (i.e., T182D and T186D, or TGIF2^DD^ for short, Fig. 5G), which presents a gain-of-function mutation at the MAP kinase sites of TGIF2. We constructed stable H1299 cell lines overexpressing TGIF2^DD^ and silenced HDAC1 in TGIF2^DD^-overexpressing cells. The ectopic expression of TGIF2^DD^ significantly decreased and increased the expression of E-cadherin and vimentin, respectively, which was rescued by HDAC1 knockdown (Fig. 5H, I). Moreover, the increased migration capacity of p-TGIF2-expressing H1299 cells were also repressed by HDAC1 knockdown (Fig. 5J, K). These results indicate that the phosphorylation of TGIF2 recruits HDAC1 to repress the transcription of E-cadherin, which promotes EMT and metastasis in LUAD.

### TGIF2-enhanced LUAD metastasis is inhibited by dual inhibition of ERK and HDAC1

**Fig. 6:**
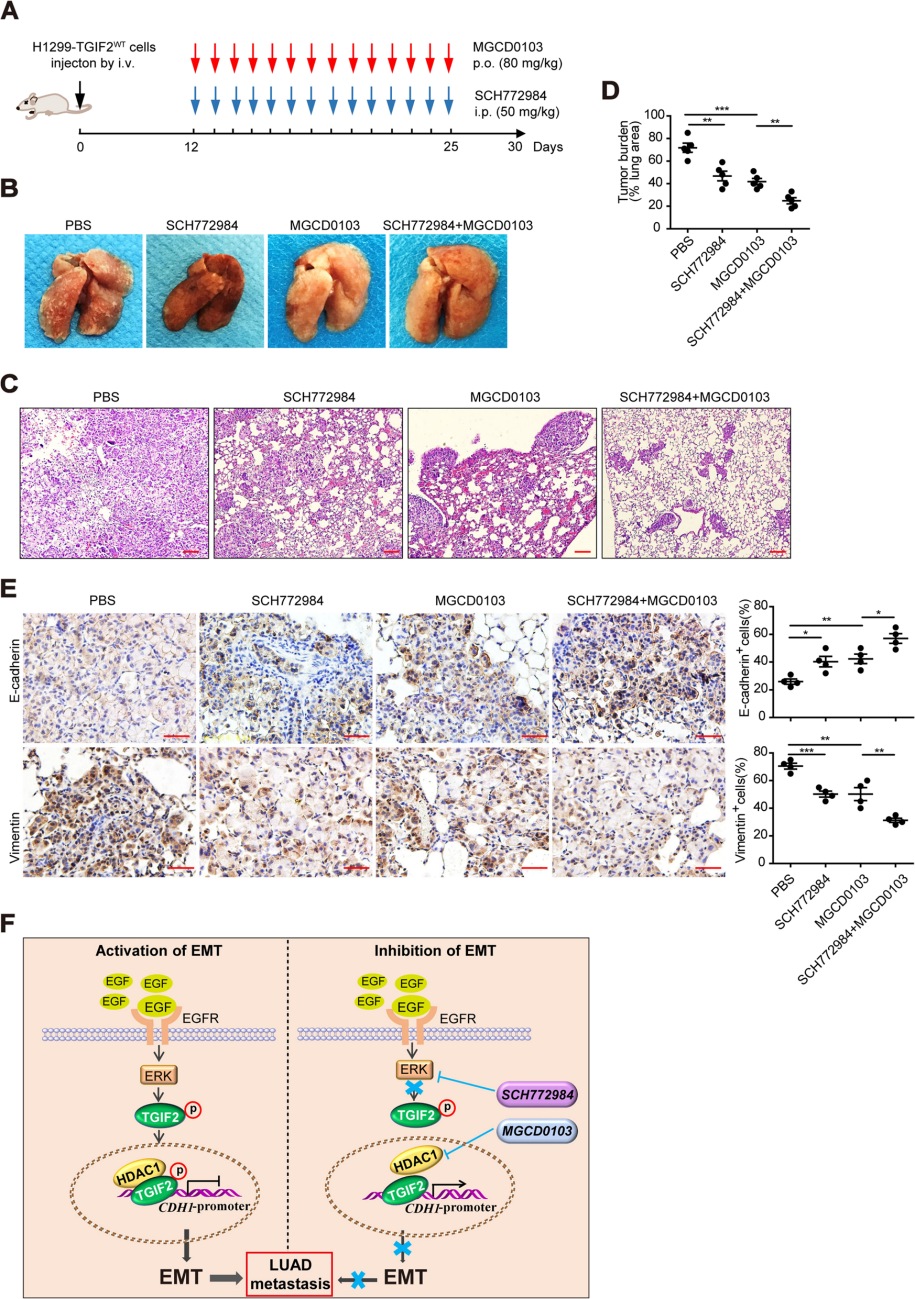
TGIF2-enhanced LUAD metastasis is inhibited by dual inhibition of ERK and HDAC1. (**A**) Schematic procedure of the in vivo mouse xenograft experiment. Mice were intravenously injected with 2 × 10^6^ TGIF2^WT^-overexpressing H1299 cells. After 12 d, mice were intraperitoneally injected with 50 mg/kg ERK inhibitor (SCH772984) and/or not intragastrically administered with 80 mg/kg HDAC1 inhibitor (MGCD0103) for 14 d. PBS was used as a control. (**B**) Representative lung images of different groups after inoculation for 30 d. (**C**) H&E staining analysis of lung metastasis in the indicated mice. Scale bars, 250 μm. (**D**) Percentage of metastatic area in mouse lungs. (**E**) IHC of E-cadherin and Vimentin expression in the indicated lung tumors (*n* = 4). Scale bars, 50 μm. (**F**) Proposed model illustrating the role of p-TGIF2 in promoting EMT and metastasis. In LUAD, activation of EGF/EGFR/ERK signaling phosphorylates TGIF2, which recruits HDAC1 and downregulates CDH1 transcription. However, dual inhibition of p-TGIF2 and HDAC1 represses the increased EMT by the p-TGIF2/HDAC1 complex. *, *p* < 0.05; **, *p* < 0.01; ***, *p* < 0.001. All data are representative of three repeated experiments

To explore whether the inhibition of p-TGIF2 and HDAC1 can repress the exacerbated metastasis of TGIF2-overexpressing (TGIF2^high^) cells, we constructed an intravenous mouse xenograft model using TGIF2-overexpressing H1299 cells; PBS, ERK inhibitor (SCH772984), HDAC1 inhibitor (MGCD0103), or ERK inhibitor plus HDAC1 inhibitor were administrated to different groups of mice (Fig. 6A). We observed decreased tumor metastasis with either administration of ERK or HDAC1 inhibitor. Importantly, dual inhibition of ERK and HDAC1 significantly ameliorated TGIF2^high^ cell-induced metastasis (Fig. 6B), which was confirmed based on the tumor burden with H&E staining (Fig. 6C, D). In addition, we observed increased expression of E-cadherin in mouse lungs treated with ERK inhibitor or HDAC1 inhibitor, which was further enhanced by dual inhibition of ERK and HDAC1 (Fig. 6E). These results suggest that the dual inhibition of p-TGIF2 and HDAC1 can effectively repress metastasis in TGIF2-overexpressing LUAD cells, and thus may be a promising therapeutic strategy.

## Discussion

In the present study, we demonstrated that TGIF2 played a key role in regulating EMT and metastasis in LUAD. Phosphorylation of TGIF2 by EGFR/ERK signaling recruited HDAC1 and repressed E-cadherin transcription, resulting in EMT and metastasis. Accordingly, the dual inhibition of TGIF2 phosphorylation and HDAC1 repressed TGIF2-enhanced EMT and metastasis (Fig. 6F).

TGIF2 was previously shown to recruit HDACs to repress the activation of TGF-β-responsive transcription [11]. However, few studies have investigated the mechanism by which TGIF2 is involved in cancer progression. In this study, we revealed that TGIF2 recruits HDAC1 to repress E-cadherin transcription and found that the ectopic expression of TGIF2WT promoted LUAD cell migration, EMT, and metastasis, while the phosphorylation-deficient TGIF2 mutant lost these functions; therefore, TGIF2 phosphorylation as a consequence of EGFR/RAS/ERK signaling was necessary for recruiting HDAC1 and activating the EMT program. Moreover, we observed endogenous binding of p-TGIF2 with HDAC1 in LUAD cells, but not in phosphorylation-deficient TGIF2 mutant cells. Our findings indicated that the phosphorylation of TGIF2 is a key step for HDAC1 recruitment in TGIF2-promoted EMT and metastasis in LUAD.

TGIF2 has been reported to interact with PKM2 and recruit HDAC3 to the E-cadherin promoter, promoting EMT in colon cancer [25]. This indicates that TGIF2 could recruit different HDACs to repress E-cadherin expression in different cancer types, reflecting tumor heterogeneity. We previously demonstrated that TGIF2 phosphorylation promoted *OCT4* transcription in an HDAC-independent manner, because TGIF2 phosphorylation improved its stability. Furthermore, TGIF2 may exert its transcription function by cooperating with other cofactors in LUAD, which is consistent with the context-dependent activities of other TALE homeoproteins [26].

The MAPK pathway, commonly known as the RAS/RAF/MEK/ERK signal cascade, is the most frequently mutated signaling pathway in lung cancer; targeting the MAPK pathway has long been considered a promising strategy in cancer therapy [27]. However, the clinical benefits of related inhibitors are compromised by the frequent occurrence of acquired resistance. HDAC inhibition has been reported to enhance the antitumor activity of MEK inhibitors in lung cancers harboring RAS mutations [28]. Another study emphasized the active inhibitory role of HDAC and ERK signaling cascades in restricting MHC II expression in lung cancer cells, and suggested that the combinatorial blockade of these pathways may engender new responses to checkpoint therapies [29]. The present study showed that the expression of E-cadherin in LUAD was actively restricted by EGFR/ERK/TGIF2 signaling and HDAC1 expression, and dual inhibition of ERK and HDAC1 showed a greater inhibitory effect on TGIF2-induced LUAD metastasis than either treatment alone. Because of its link to immunotherapy resistance, the targeting of EMT may inhibit cancer immune suppression [30]. Based on our and previous findings, the inhibition of EMT by targeting p-TGIF2 could also improve the outcomes in cancer immunotherapy, though this requires further validation.

## Conclusions

In this study, we demonstrated that the phosphorylation of TGIF2 by EGFR/ERK signaling promoted EMT and metastasis in LUAD by recruiting HDAC1 to downregulate E-cadherin expression. Combined ERK and HDAC1 inhibition significantly repressed TGIF2-enhanced metastasis, suggesting that TGIF2-overexpressing LUAD patients could potentially be treated by p-TGIF2 and HDAC1 inhibitions.

## Supplementary Information

**Additional file 1.** 

**Additional file 2.**

## Data Availability

The datasets used and/or analysed during the current study are available in a public repository from TCGA website (http://cancergenome.nih.gov/) along with (https://portal.gdc.cancer.gov/projects/TCGA-LUAD).

## Abbreviations

TGIF2: TGF-β-induced factor homeobox 2
p-TGIF2: Phosphorylated (p)-TGIF2
LUAD: Lung adenocarcinoma
EMT: Epithelial-mesenchymal transition
ChIP: Chromatin immunoprecipitation
IHC: Immunohistochemistry
GSEA: Gene set enrichment analysis
