# Supplementary material for: Phosphorylation of TGIF2 represents a therapeutic target that drives EMT and metastasis of lung adenocarcinoma
Source: BMC Cancer. 2023 Jan 16;23:52. doi: 10.1186/s12885-023-10535-9 (PMC9841675; doi:10.1186/s12885-023-10535-9)
Supplement: Supplementary file 1 — Additional file 1. [file 12885_2023_10535_MOESM1_ESM.pdf]

Figure 1D

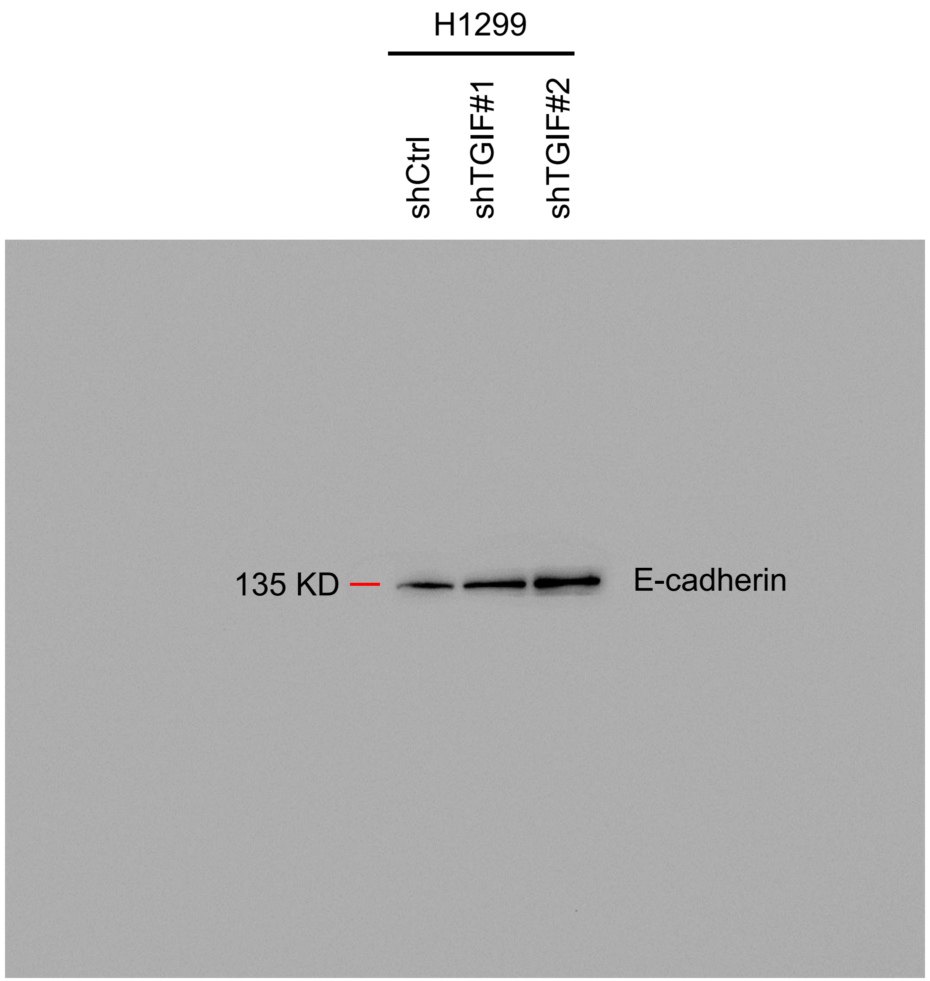
H1299
shCtrl
shTGIF#1
shTGIF#2
135 KD
E-cadherin

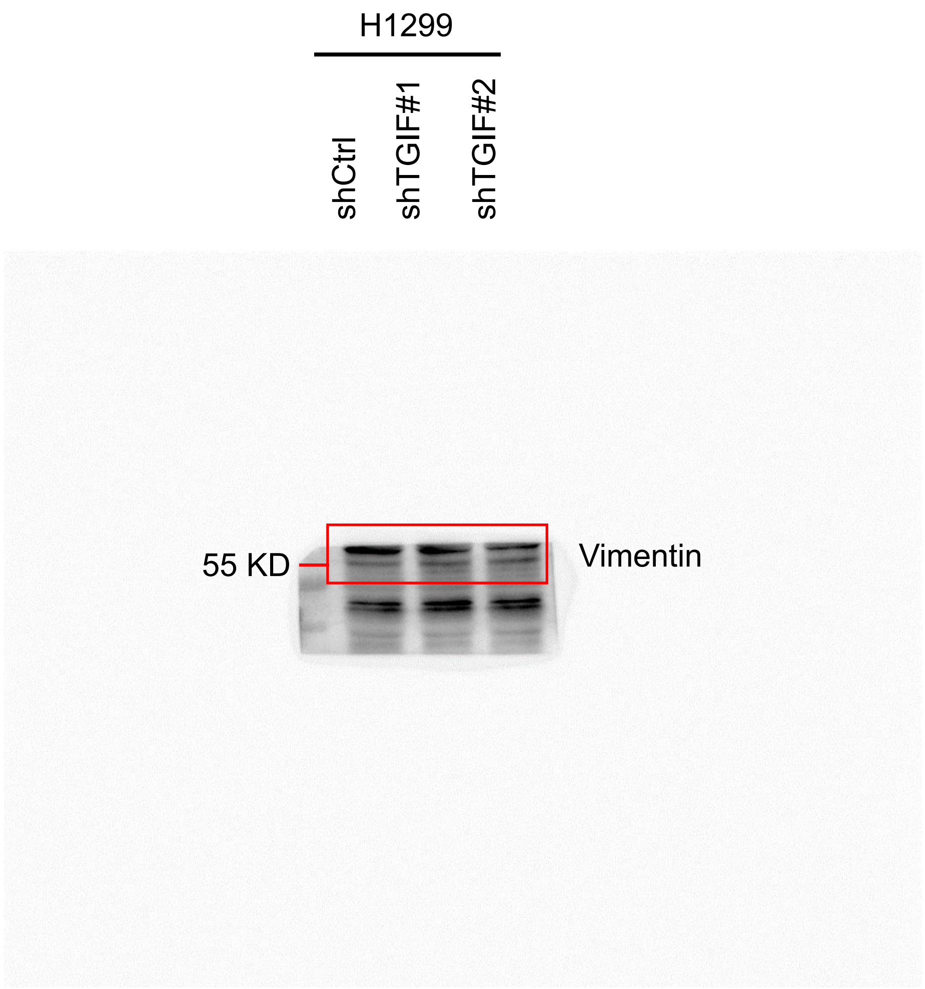
H1299
shCtrl
shTGIF#1
shTGIF#2
55 KD
Vimentin

Figure 1D

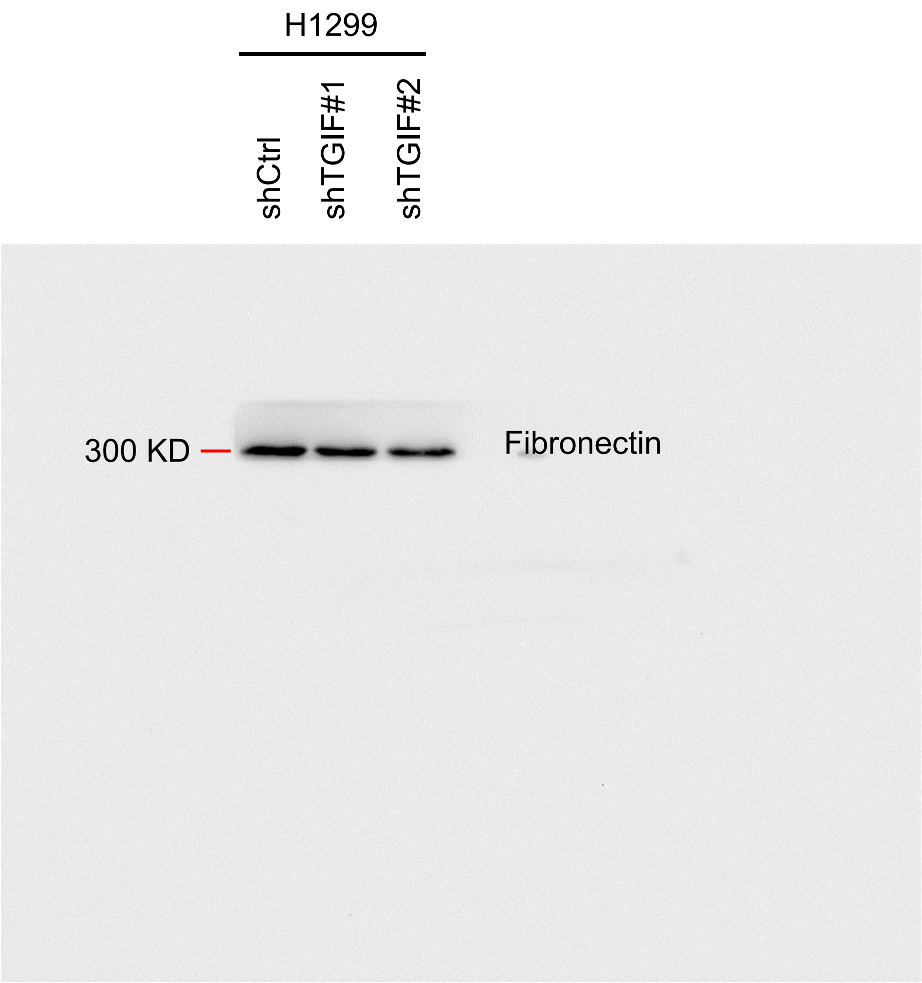
H1299
shCtrl
shTGIF#1
shTGIF#2
300 KD
Fibronectin

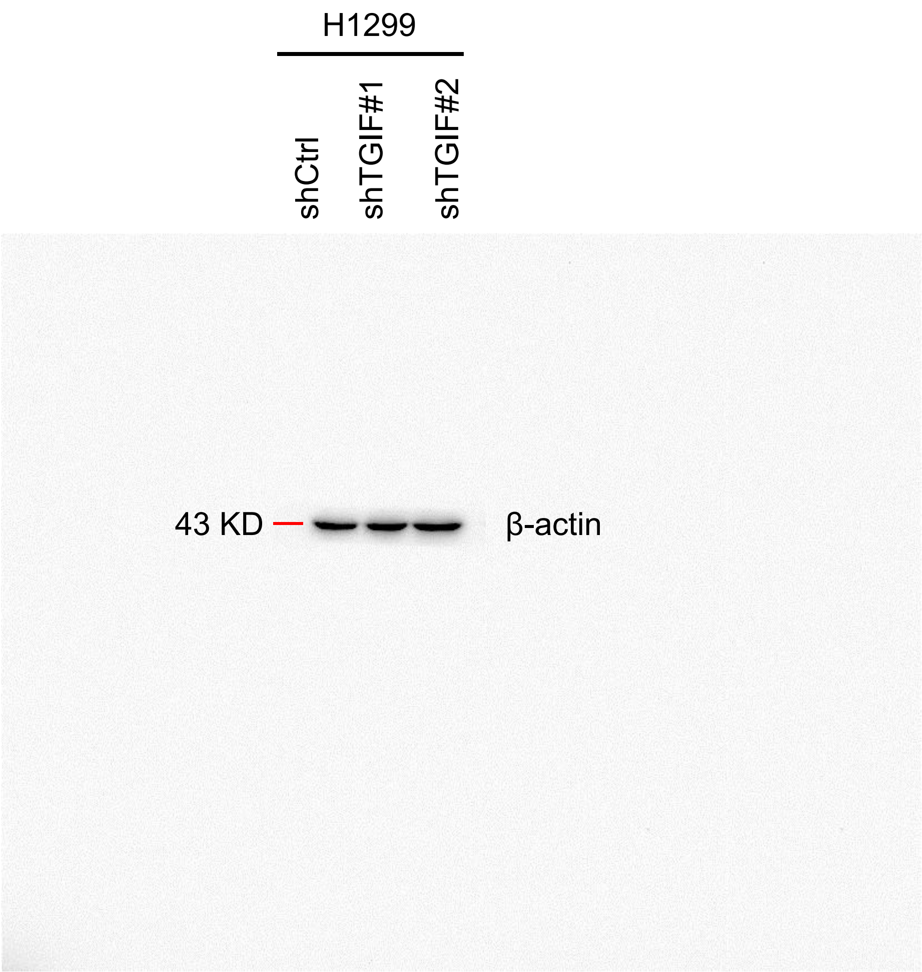
H1299
shCtrl
shTGIF#1
shTGIF#2
43 KD
β-actin

Figure 1D

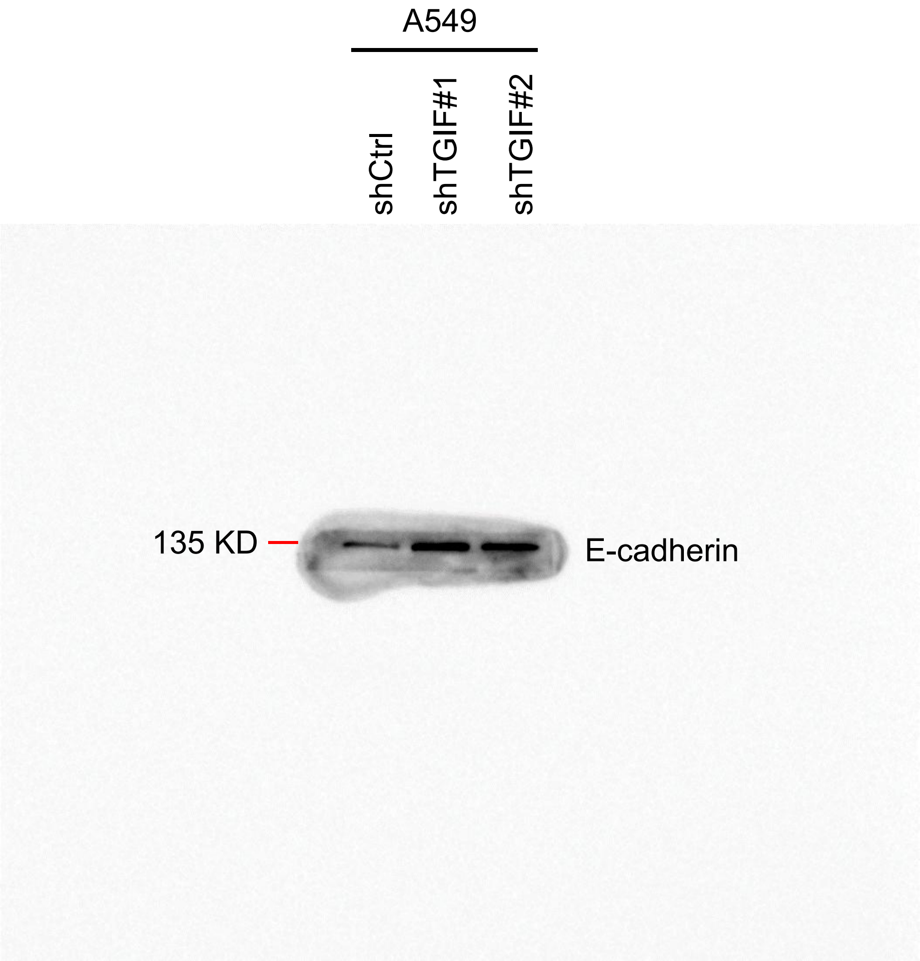
A549
shCtrl
shTGIF#1
shTGIF#2
135 KD
E-cadherin

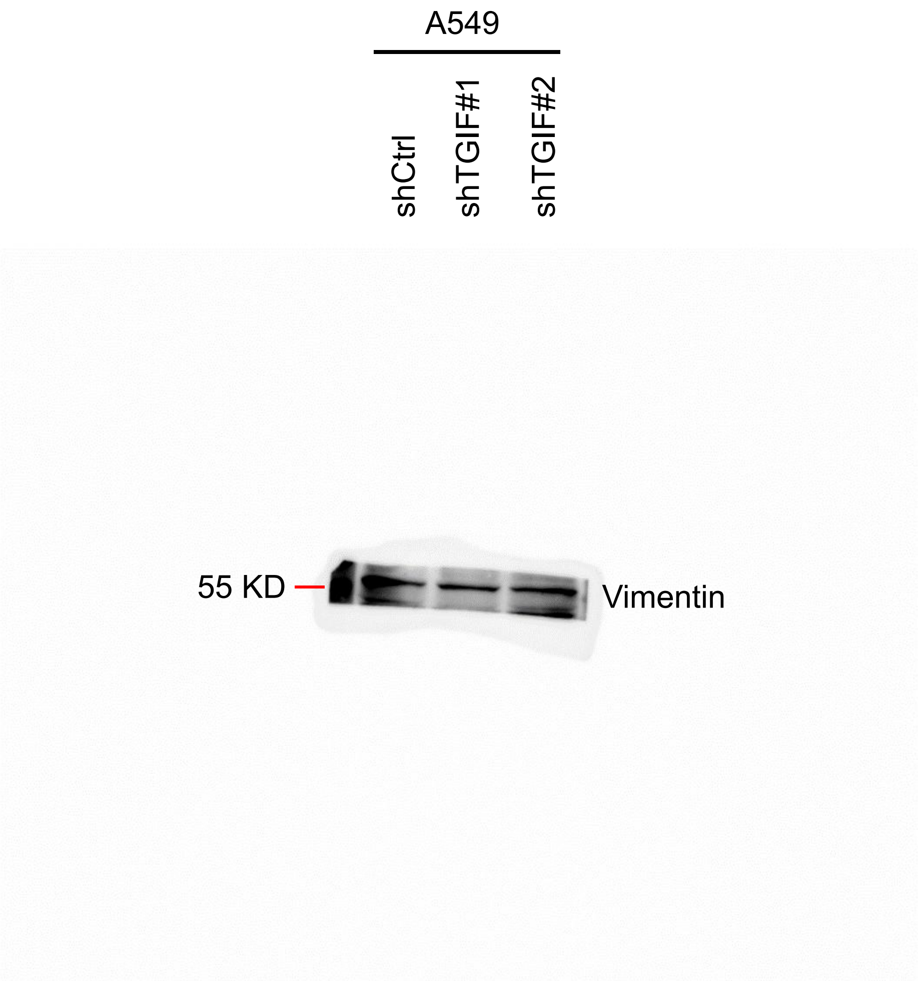
A549
shCtrl
shTGIF#1
shTGIF#2
55 KD
Vimentin

Figure 1D

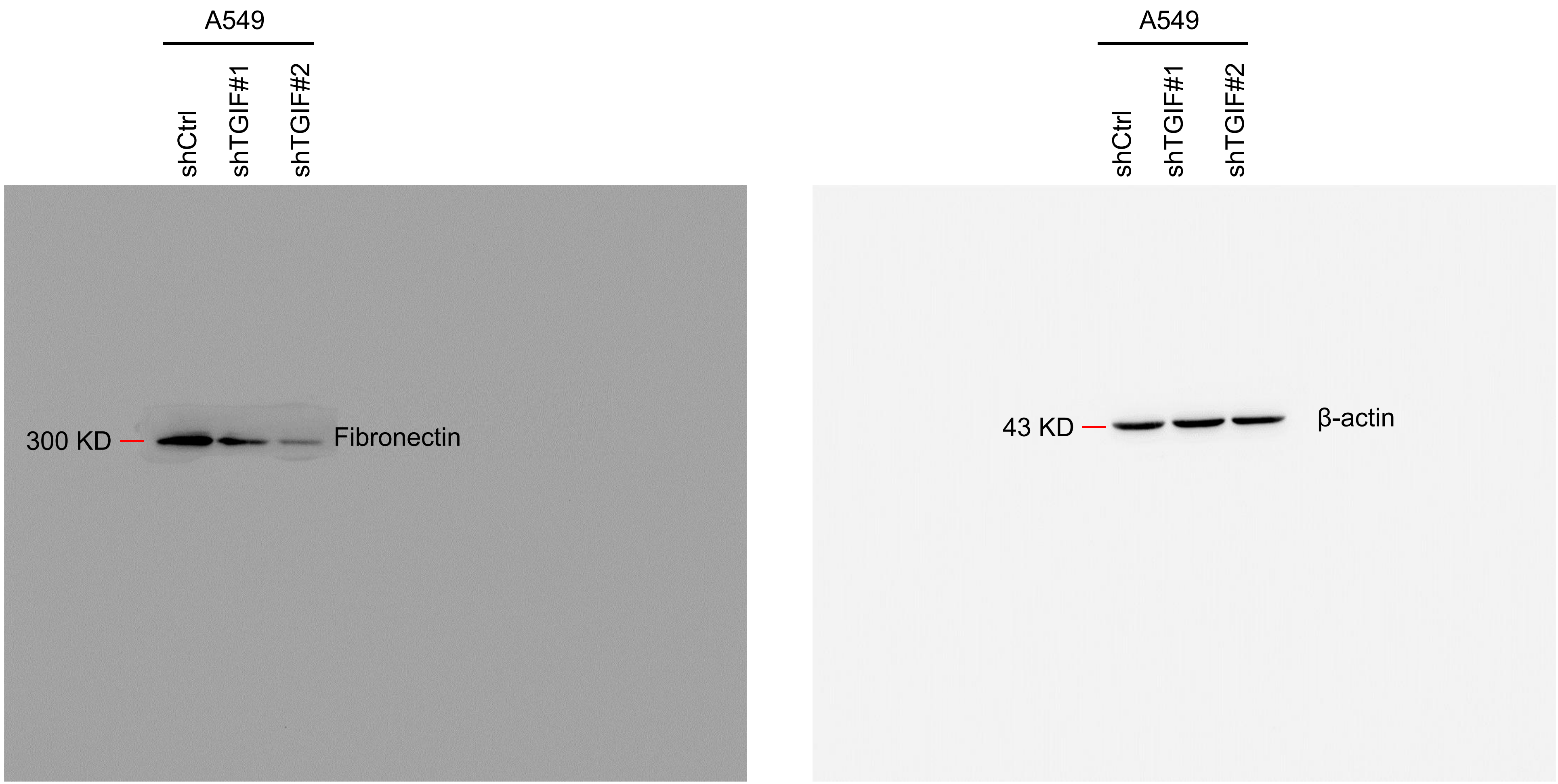
A549
shCtrl
shTGIF#1
shTGIF#2
300 KD
Fibronectin
A549
shCtrl
shTGIF#1
shTGIF#2
43 KD
β-actin

Figure 1E

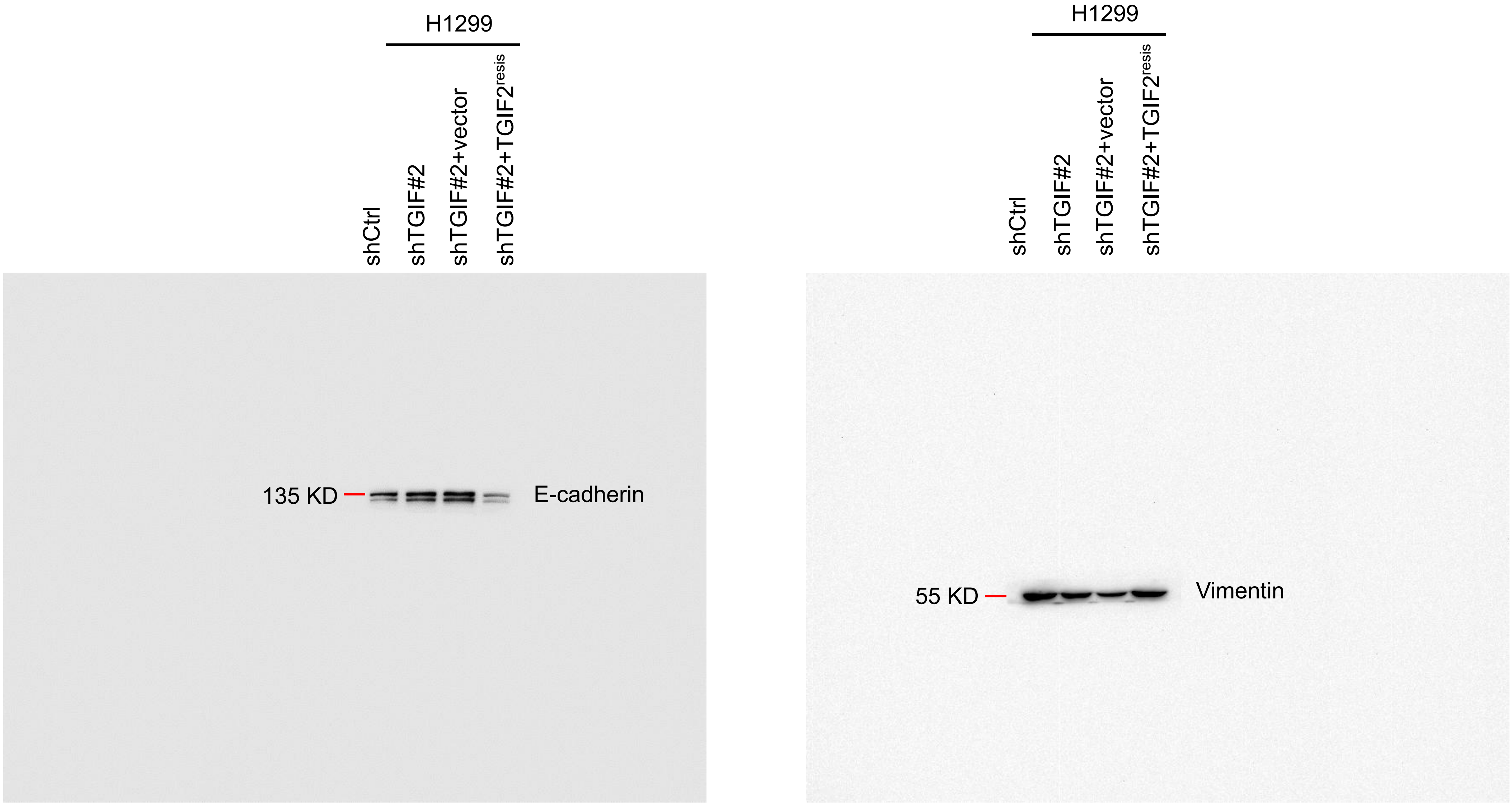
H1299
shCtrl
shTGIF#2
shTGIF#2+vector
shTGIF#2+TGIF2resis
135 KD
E-cadherin
H1299
shCtrl
shTGIF#2
shTGIF#2+vector
shTGIF#2+TGIF2resis
55 KD
Vimentin

Figure 1E

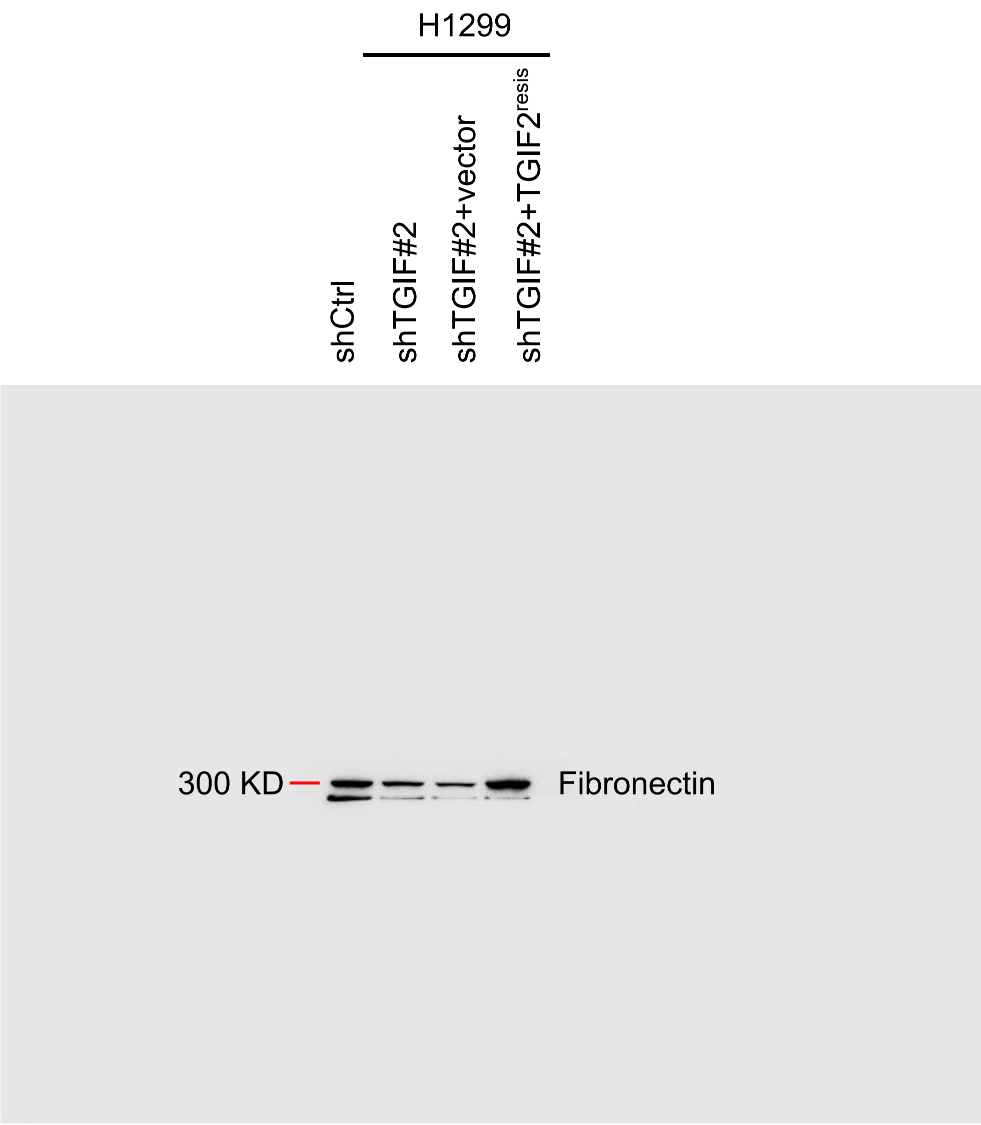
H1299
shCtrl
shTGIF#2
shTGIF#2+vector
shTGIF#2+TGIF2resis
300 KD
Fibronectin

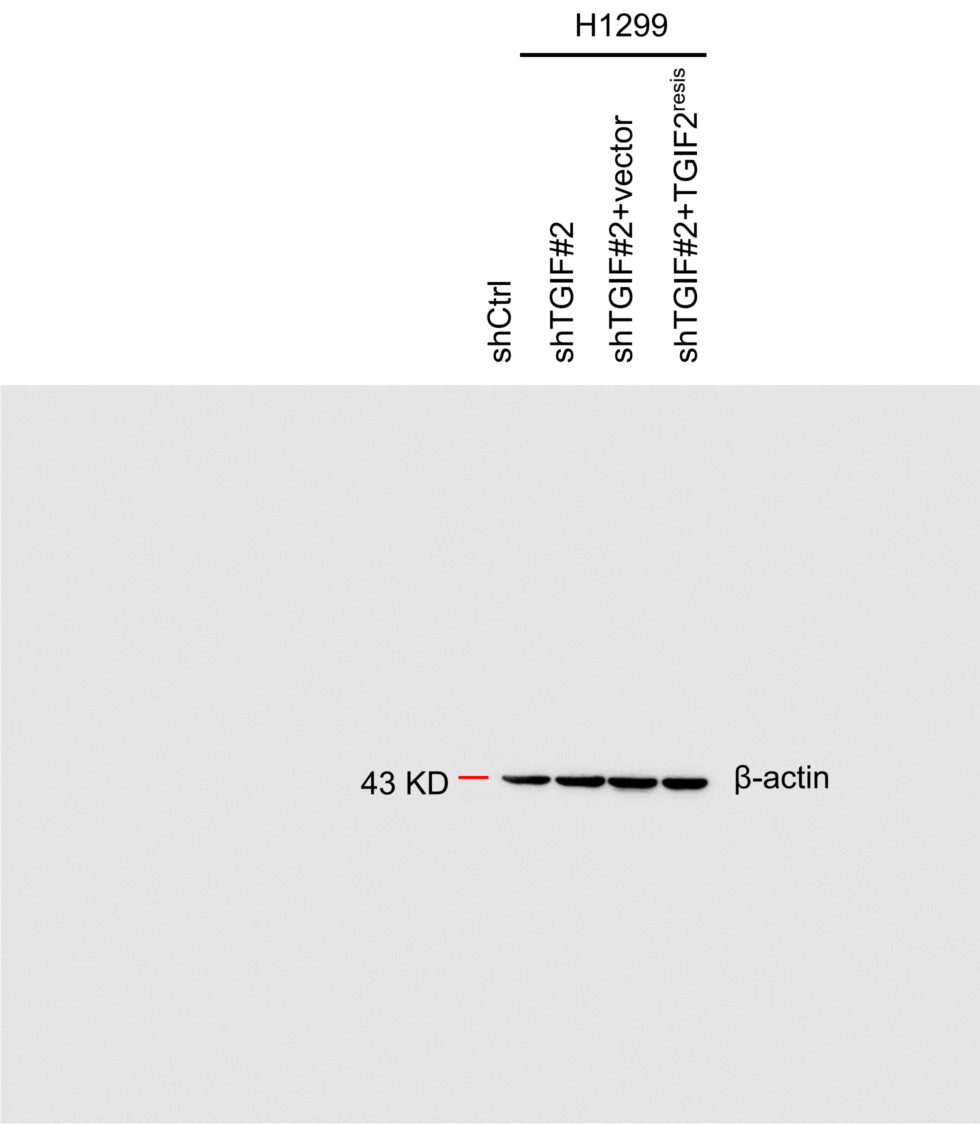
H1299
shCtrl
shTGIF#2
shTGIF#2+vector
shTGIF#2+TGIF2resis
43 KD
β-actin

Figure 3A

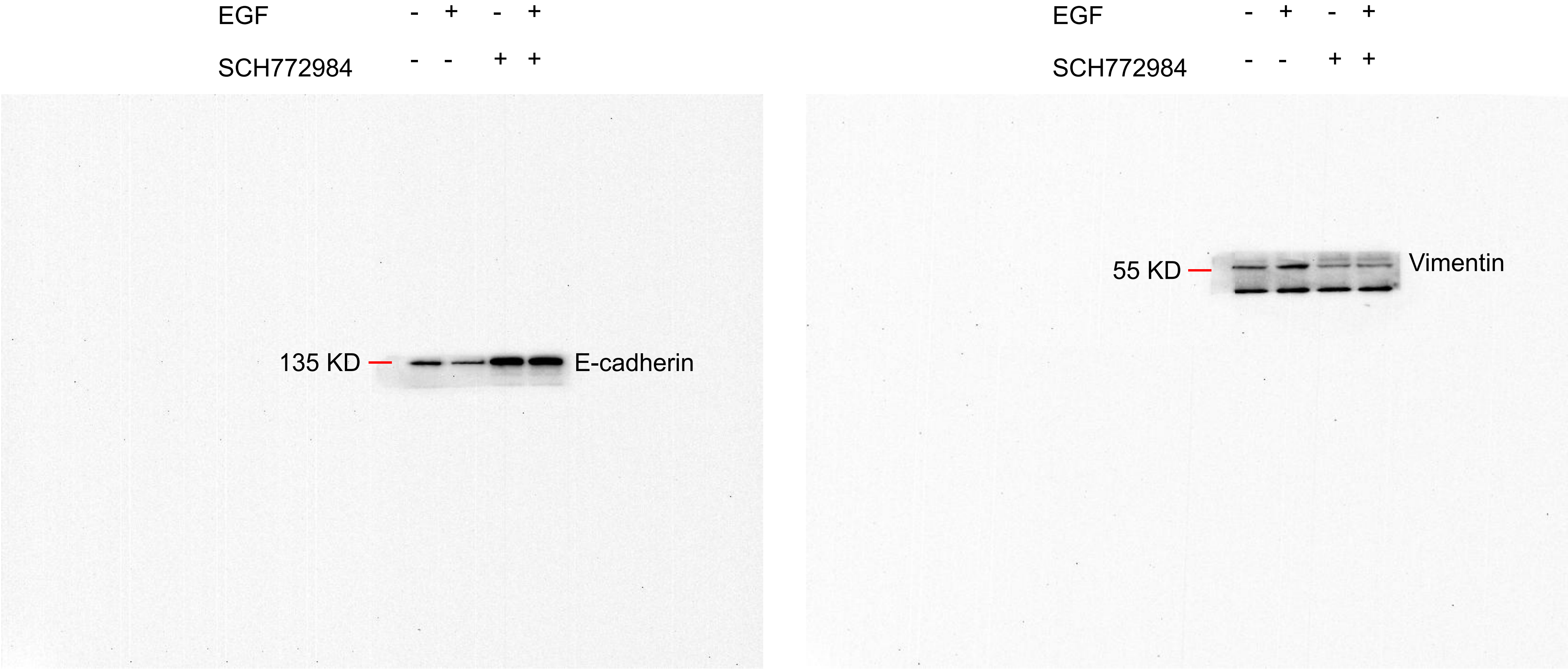
EGF
- + - +
SCH772984
- - + +
135 KD
E-cadherin
EGF
- + - +
SCH772984
- - + +
55 KD
Vimentin

Figure 3A

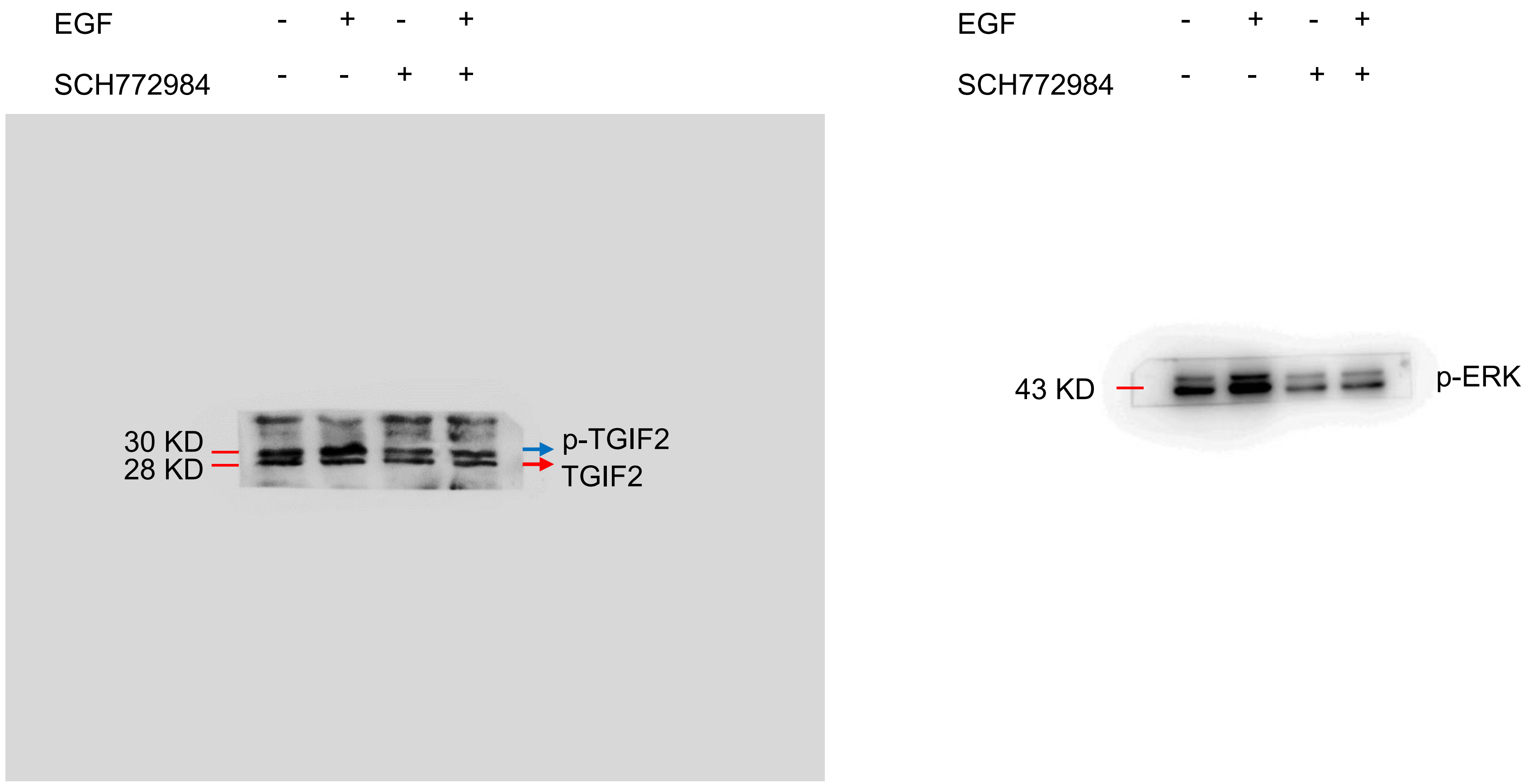

EGF - + - +
SCH772984 - - + +
30 KD
28 KD
p-TGIF2
TGIF2
EGF - + - +
SCH772984 - - + +
43 KD
p-ERK

Figure 3A

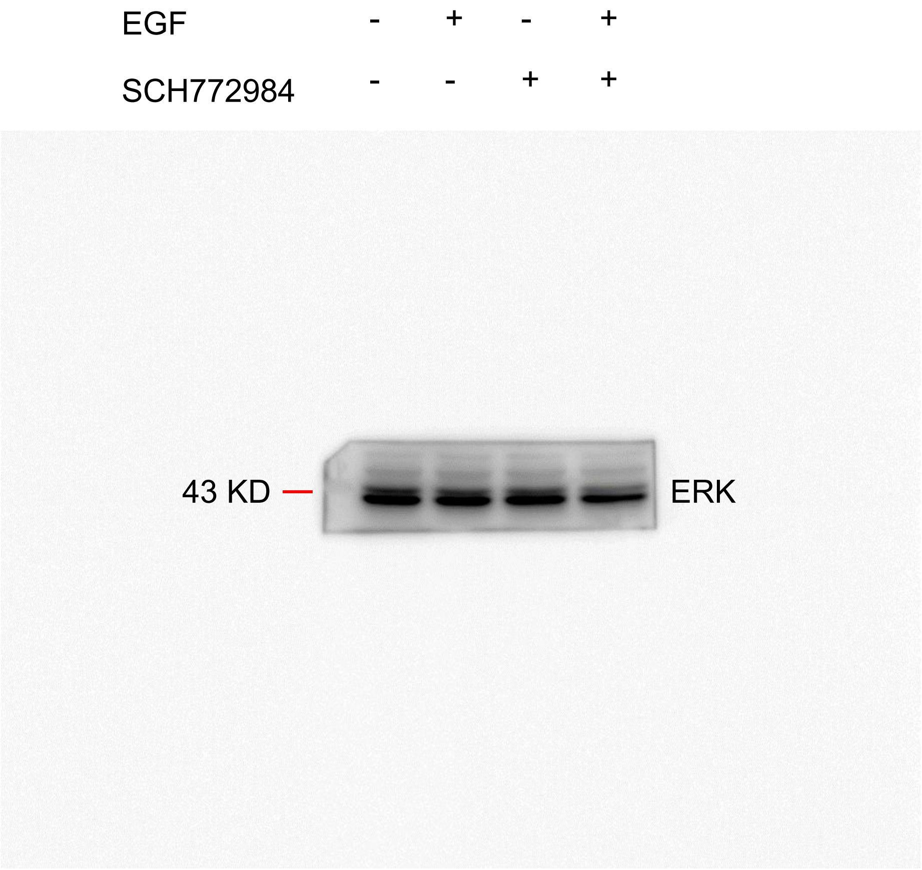

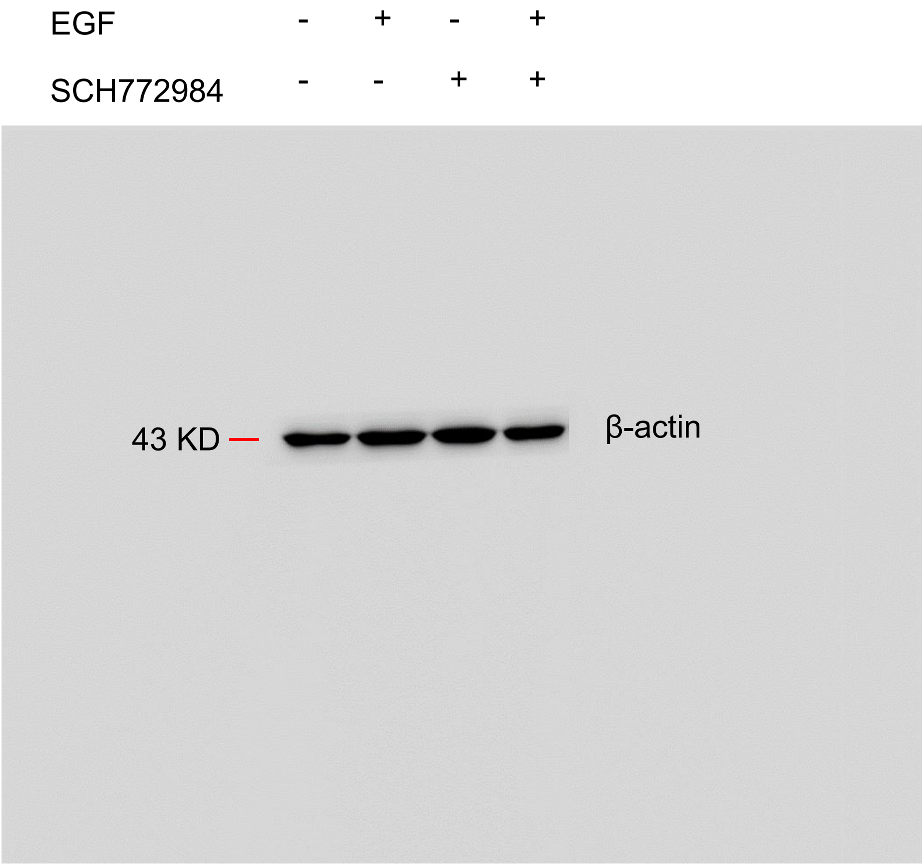

Figure 3B

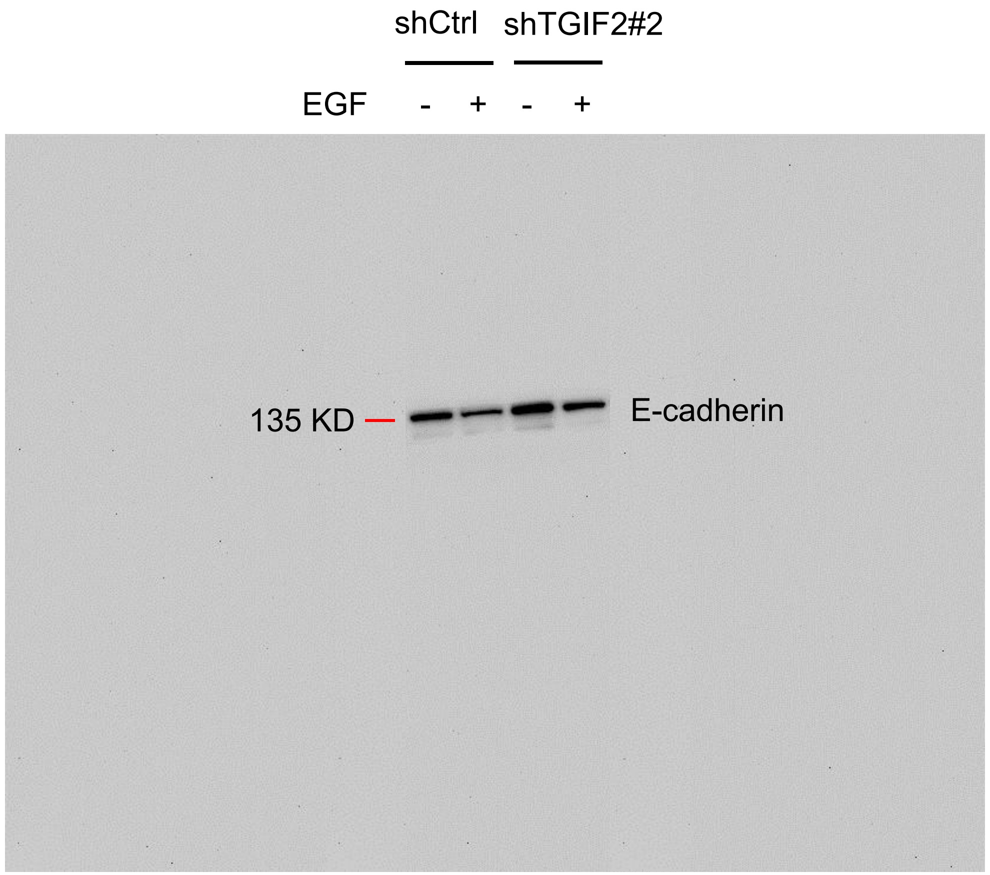

shCtrl
shTGIF2#2
EGF - + - +
135 KD
E-cadherin

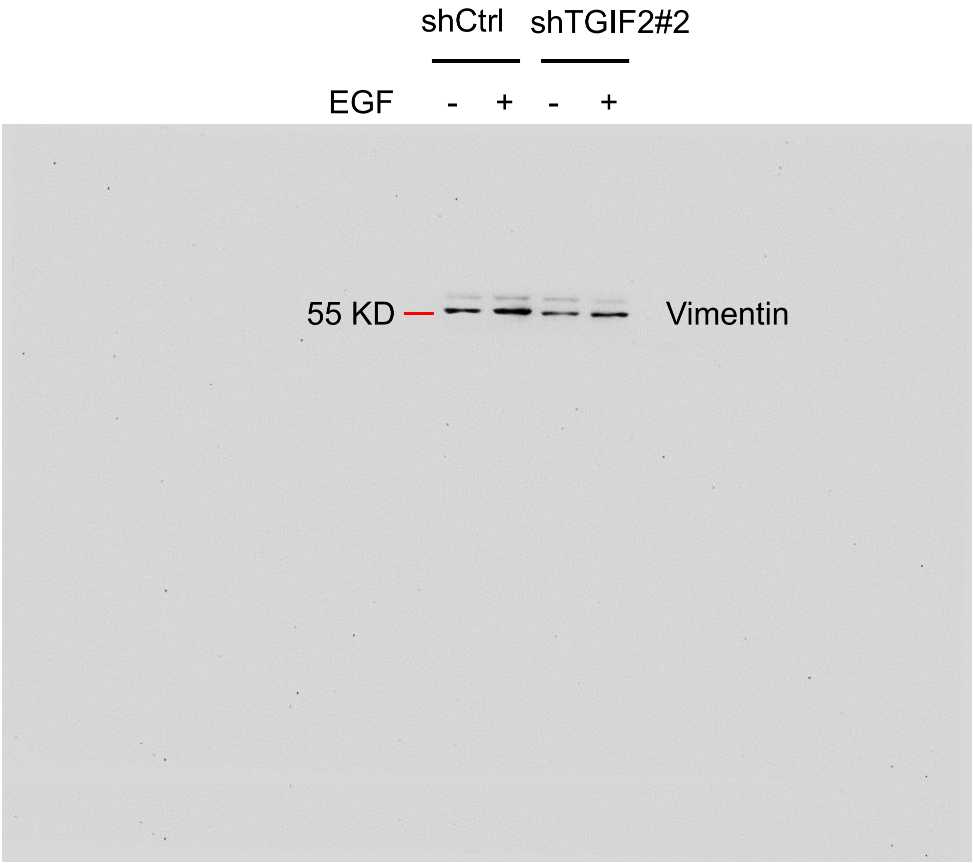

shCtrl
shTGIF2#2
EGF - + - +
55 KD
Vimentin

Figure 3B

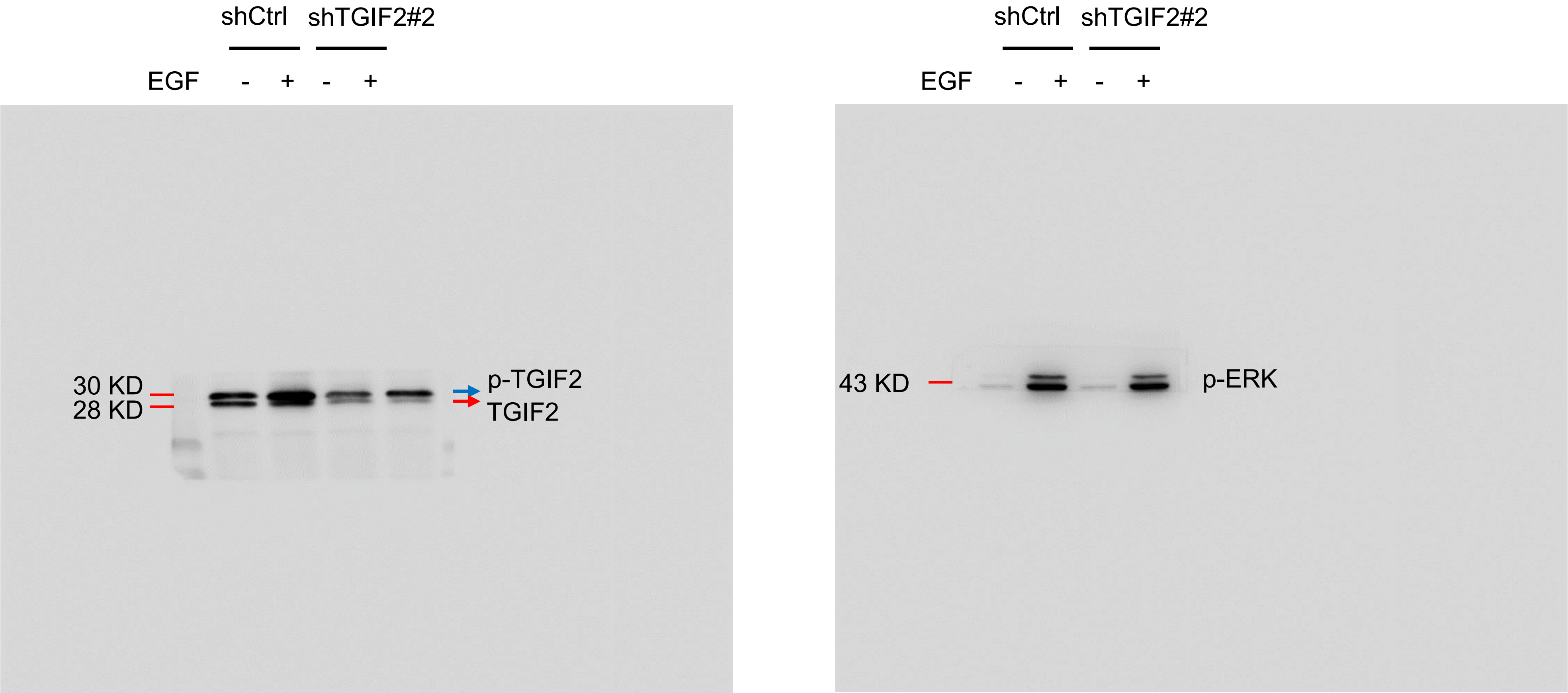

shCtrl
shTGIF2#2
EGF
-
+
-
+
30 KD
28 KD
p-TGIF2
TGIF2
shCtrl
shTGIF2#2
EGF
-
+
-
+
43 KD
p-ERK

Figure 3B

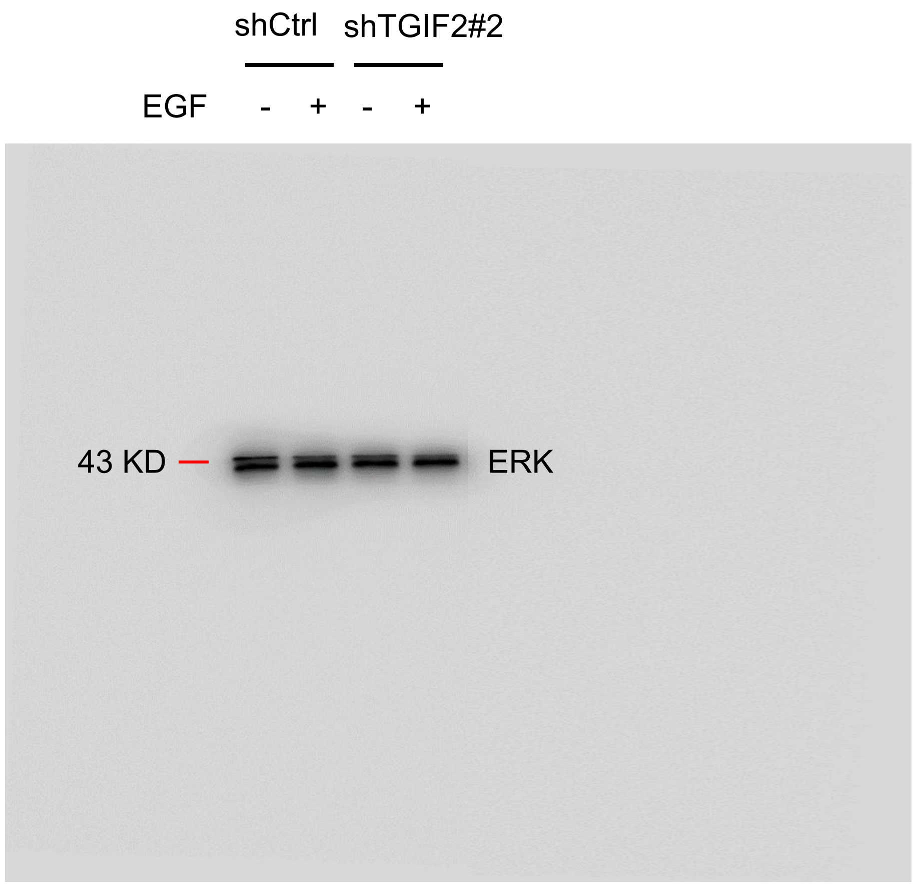
shCtrl
shTGIF2#2
EGF
-
+
-
+
43 KD
ERK

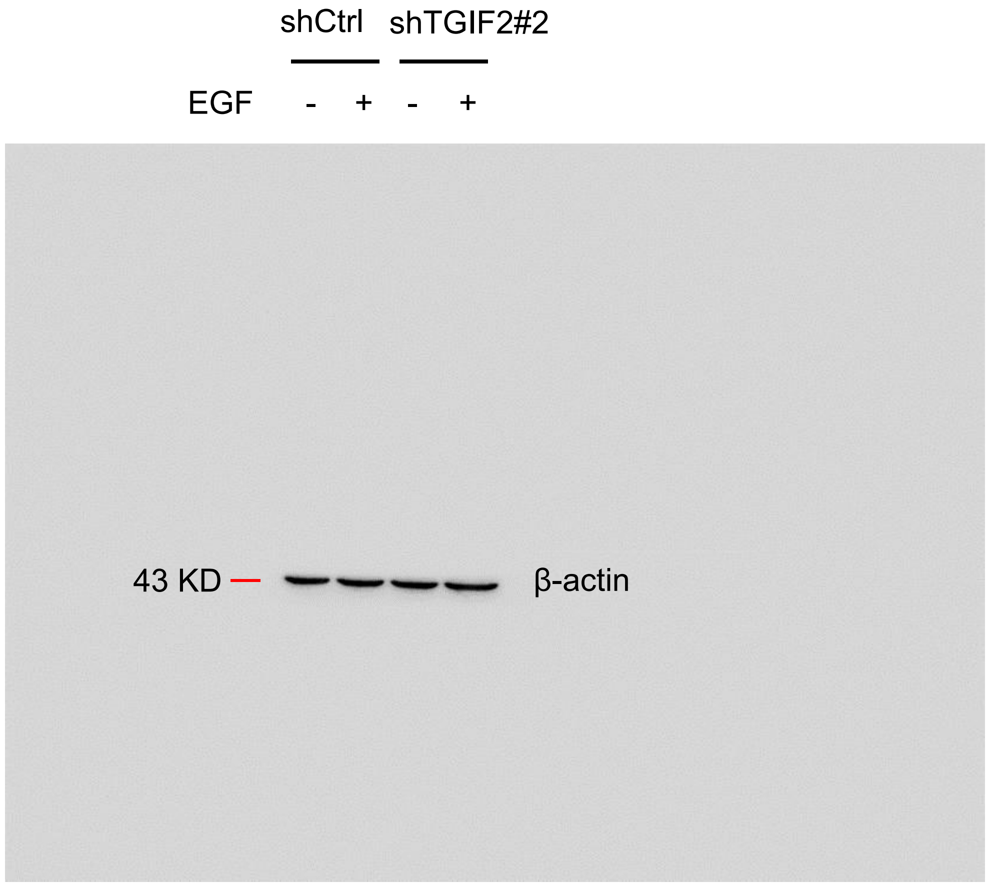
shCtrl
shTGIF2#2
EGF
-
+
-
+
43 KD
β-actin

Figure 3D

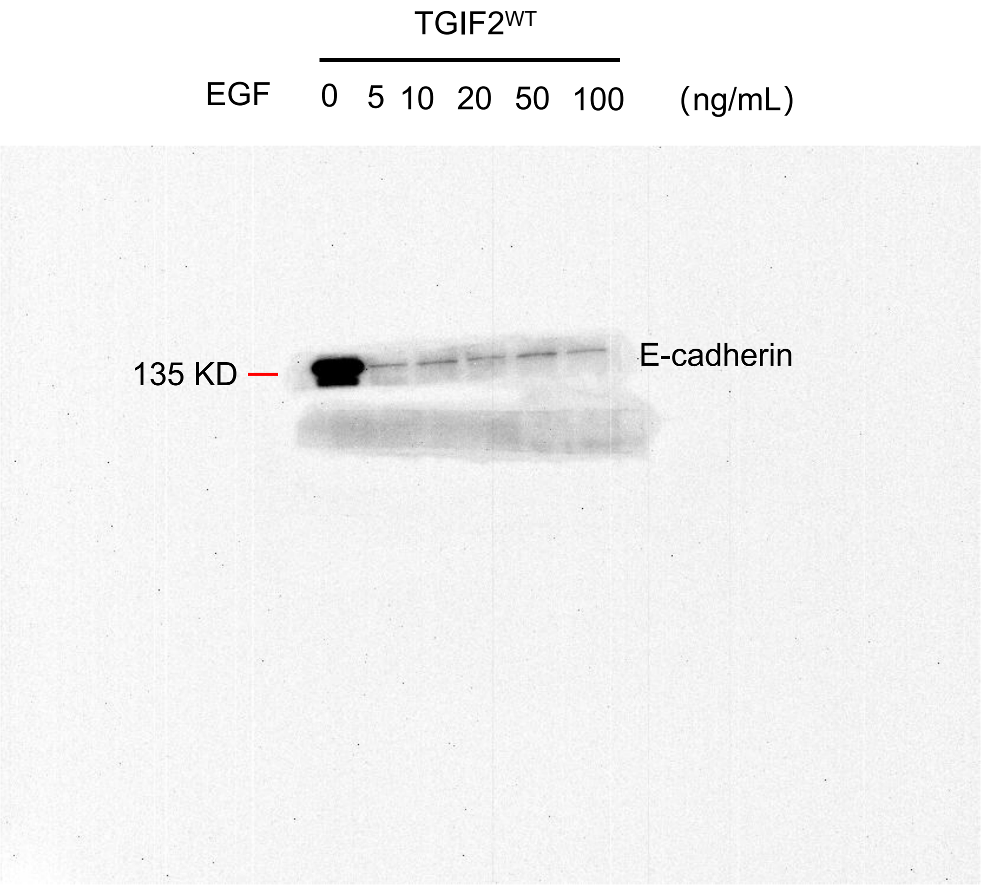

TGIF2WT
EGF 0 5 10 20 50 100 (ng/mL)
135 KD
E-cadherin

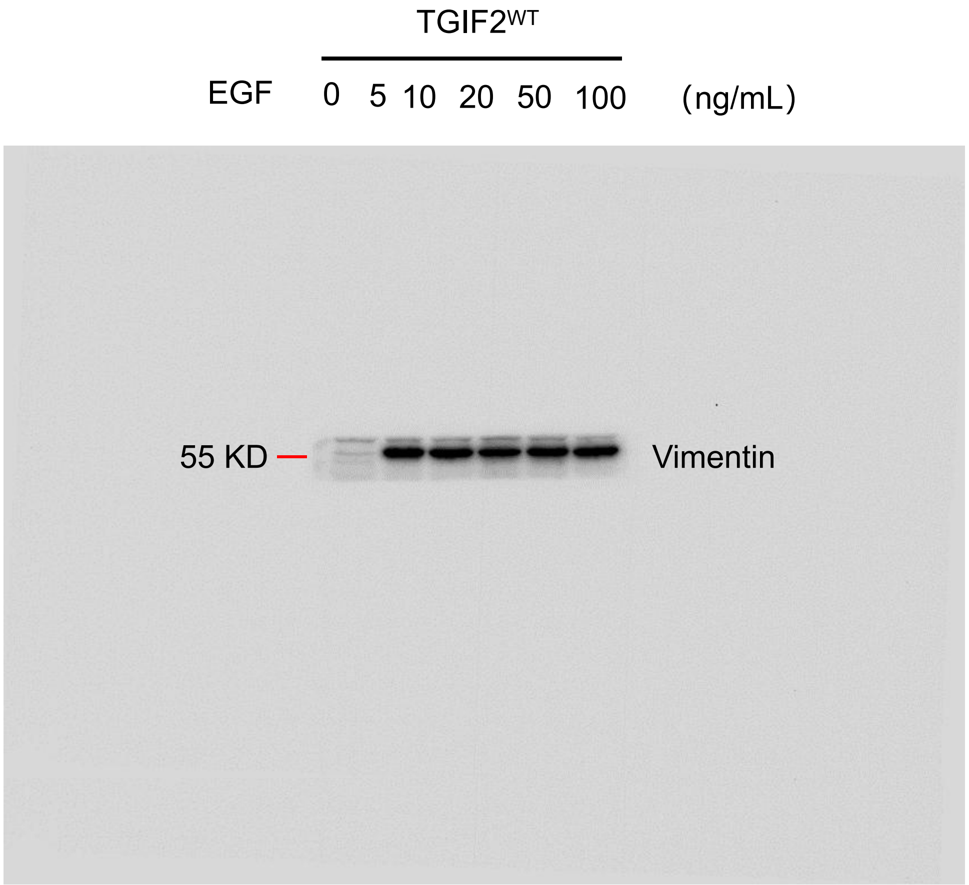

TGIF2WT
EGF 0 5 10 20 50 100 (ng/mL)
55 KD
Vimentin

Figure 3D

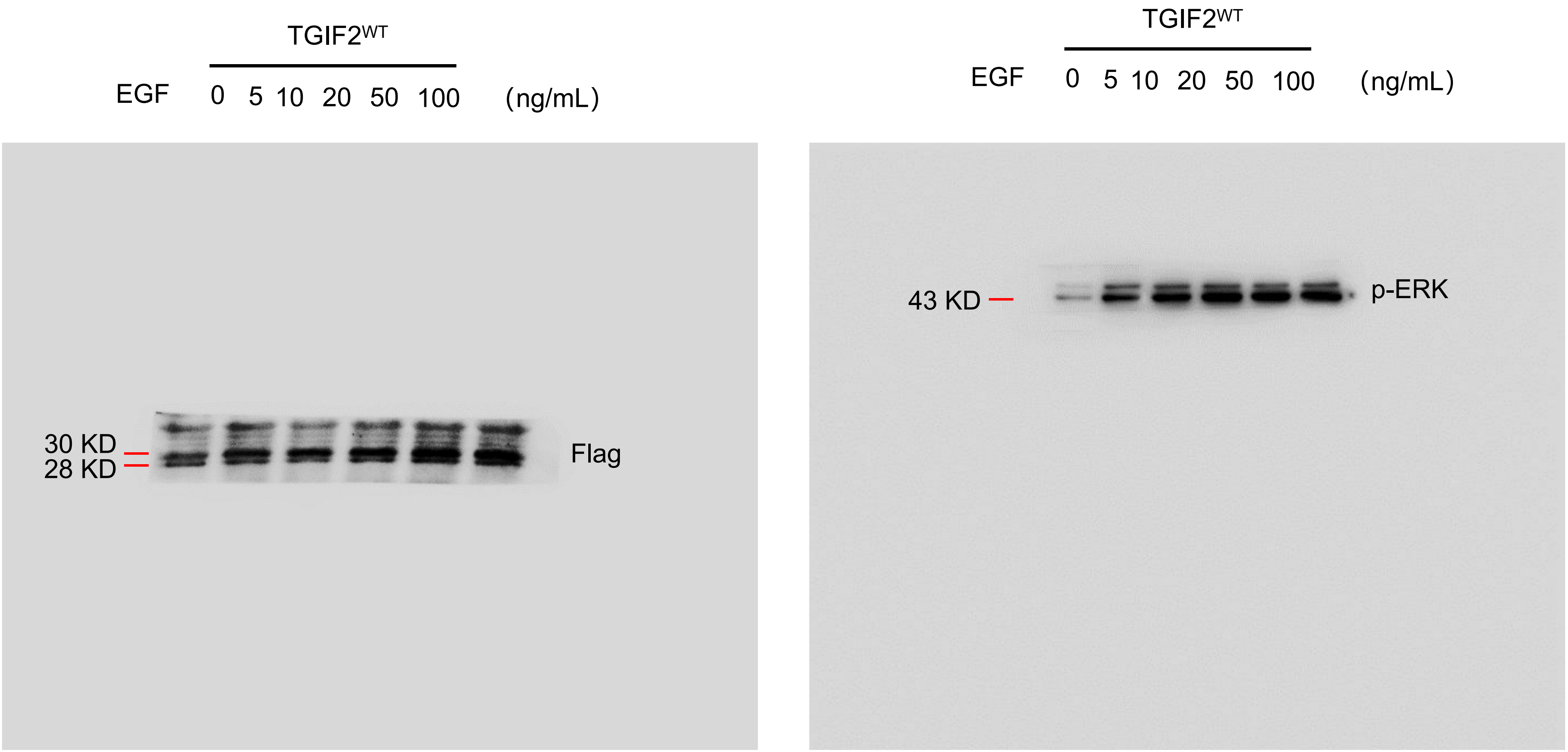
TGIF2WT
EGF
0
5
10
20
50
100
(ng/mL)
30 KD
28 KD
Flag
TGIF2WT
EGF
0
5
10
20
50
100
(ng/mL)
43 KD
p-ERK

Figure 3D

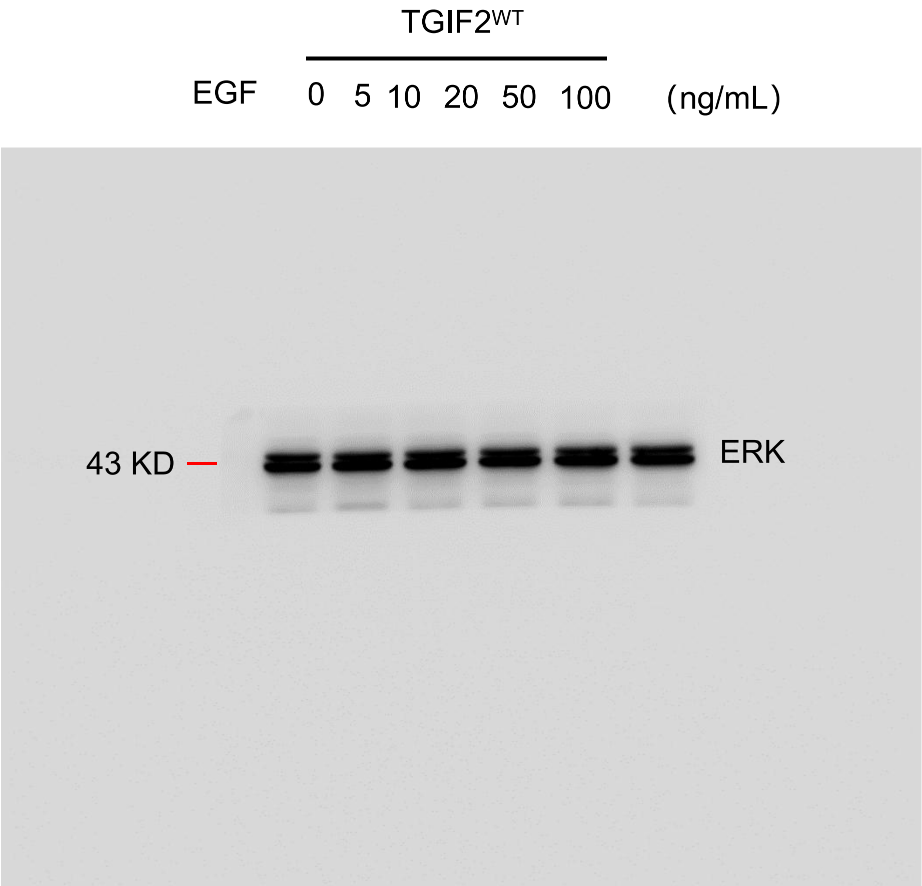
TGIF2WT
EGF 0 5 10 20 50 100 (ng/mL)
43 KD
ERK

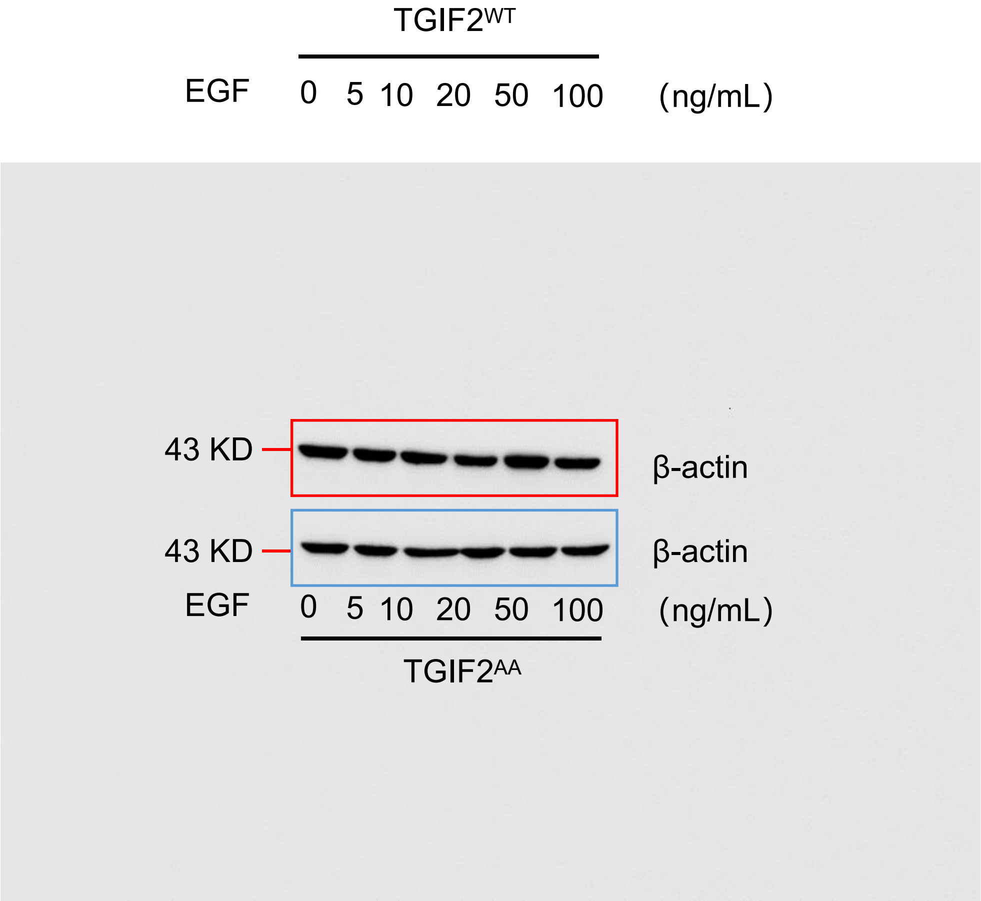
TGIF2WT
EGF 0 5 10 20 50 100 (ng/mL)
43 KD
β-actin
43 KD
β-actin
EGF 0 5 10 20 50 100 (ng/mL)
TGIF2AA

Figure 3D

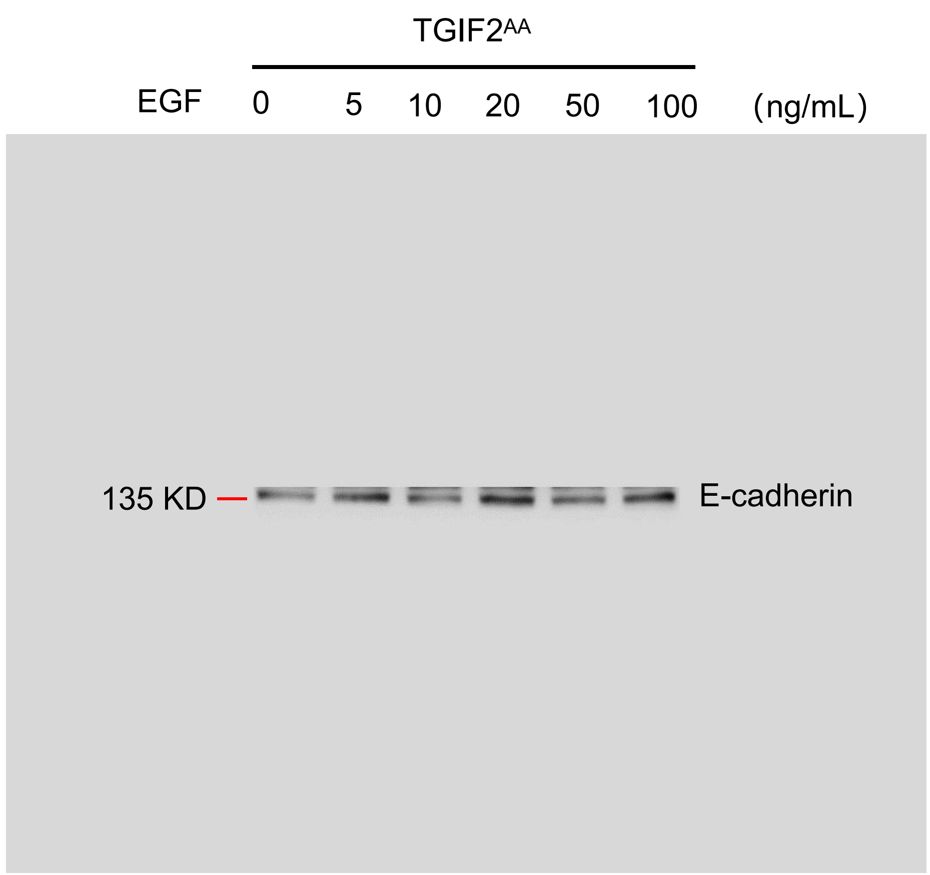
TGIF2AA
EGF 0 5 10 20 50 100 (ng/mL)
135 KD
E-cadherin

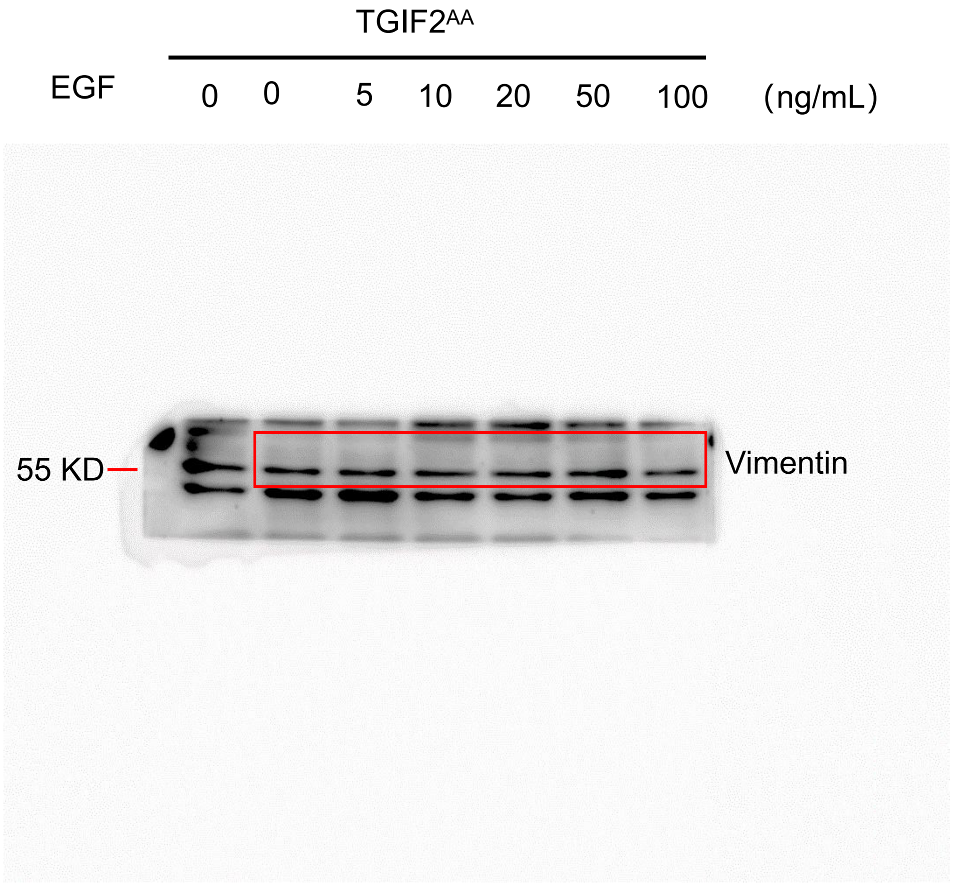
TGIF2AA
EGF 0 0 5 10 20 50 100 (ng/mL)
55 KD
Vimentin

Figure 3D

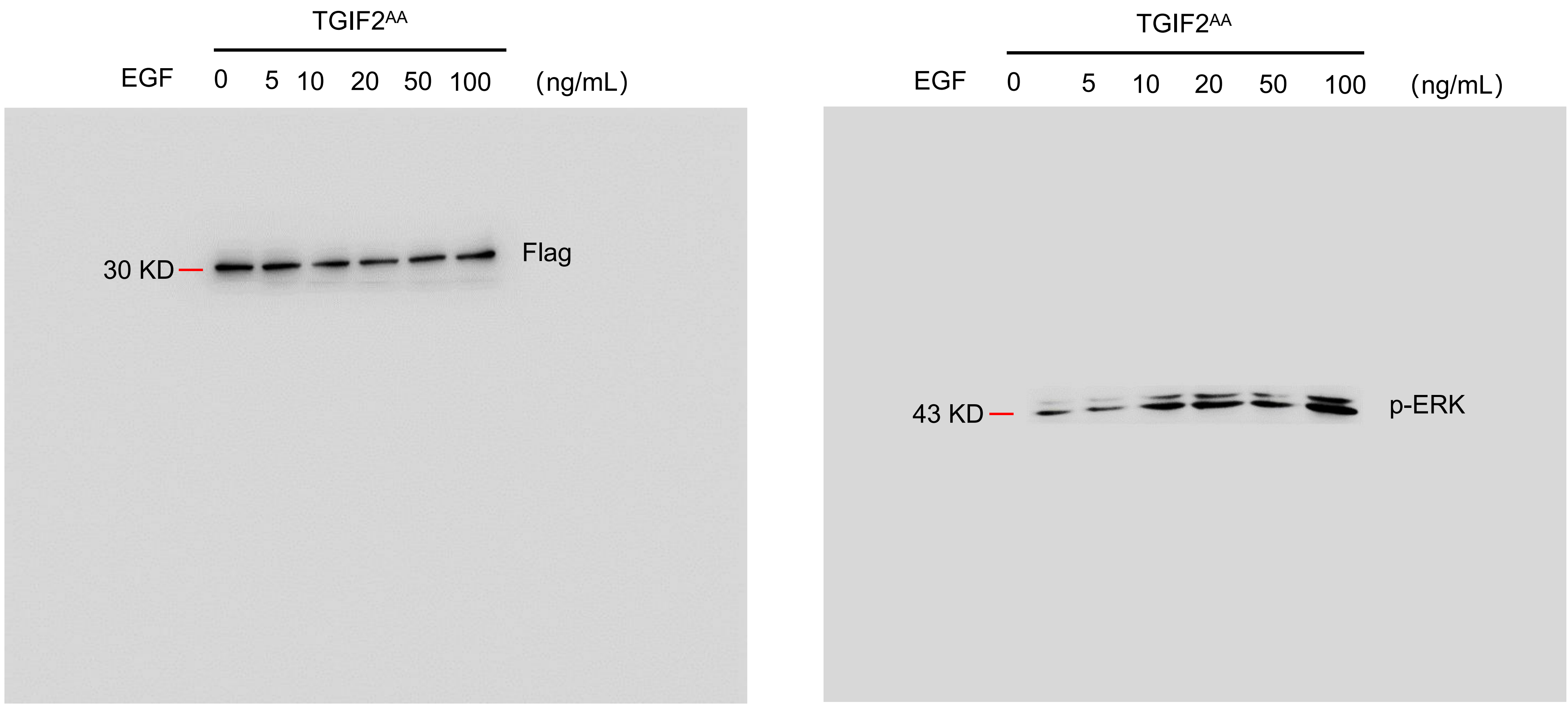
TGIF2AA
EGF 0 5 10 20 50 100 (ng/mL)
30 KD
Flag
TGIF2AA
EGF 0 5 10 20 50 100 (ng/mL)
43 KD
p-ERK

Figure 3D

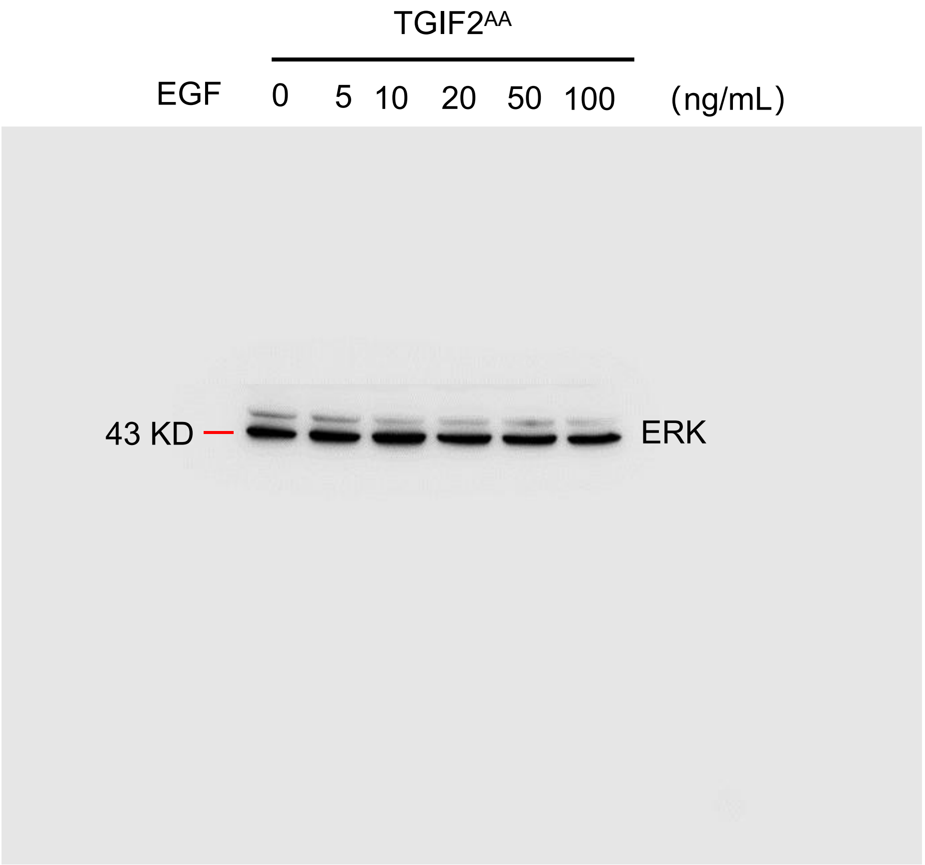
TGIF2AA
EGF 0 5 10 20 50 100 (ng/mL)
43 KD
ERK

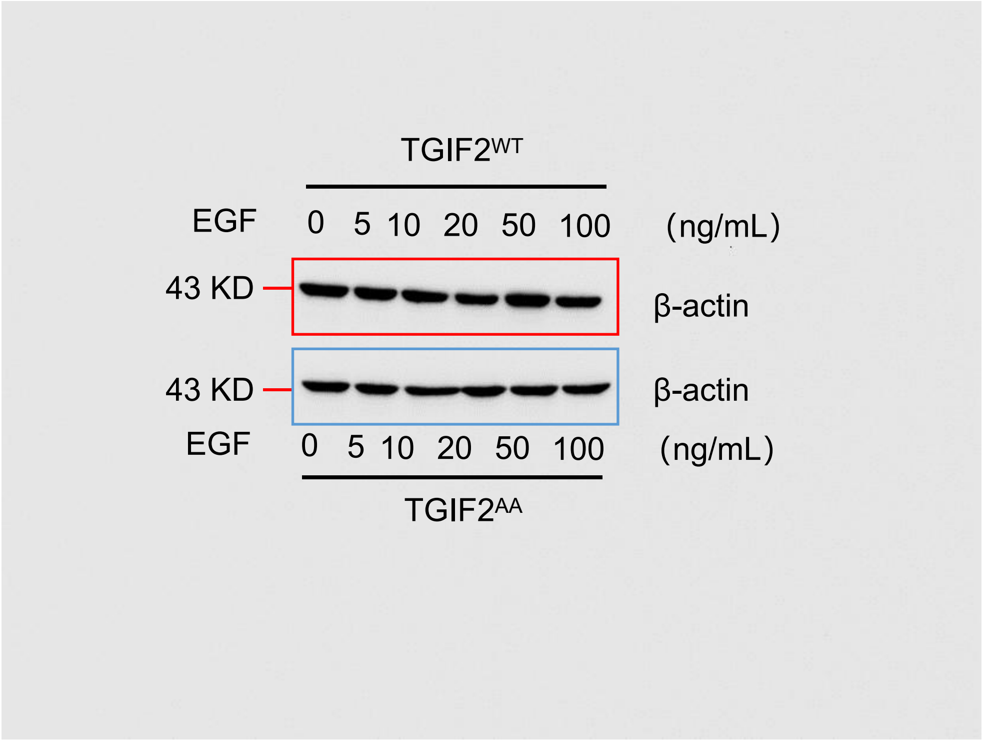
TGIF2WT
EGF 0 5 10 20 50 100 (ng/mL)
43 KD
β-actin
43 KD
β-actin
EGF 0 5 10 20 50 100 (ng/mL)
TGIF2AA

Figure 3E

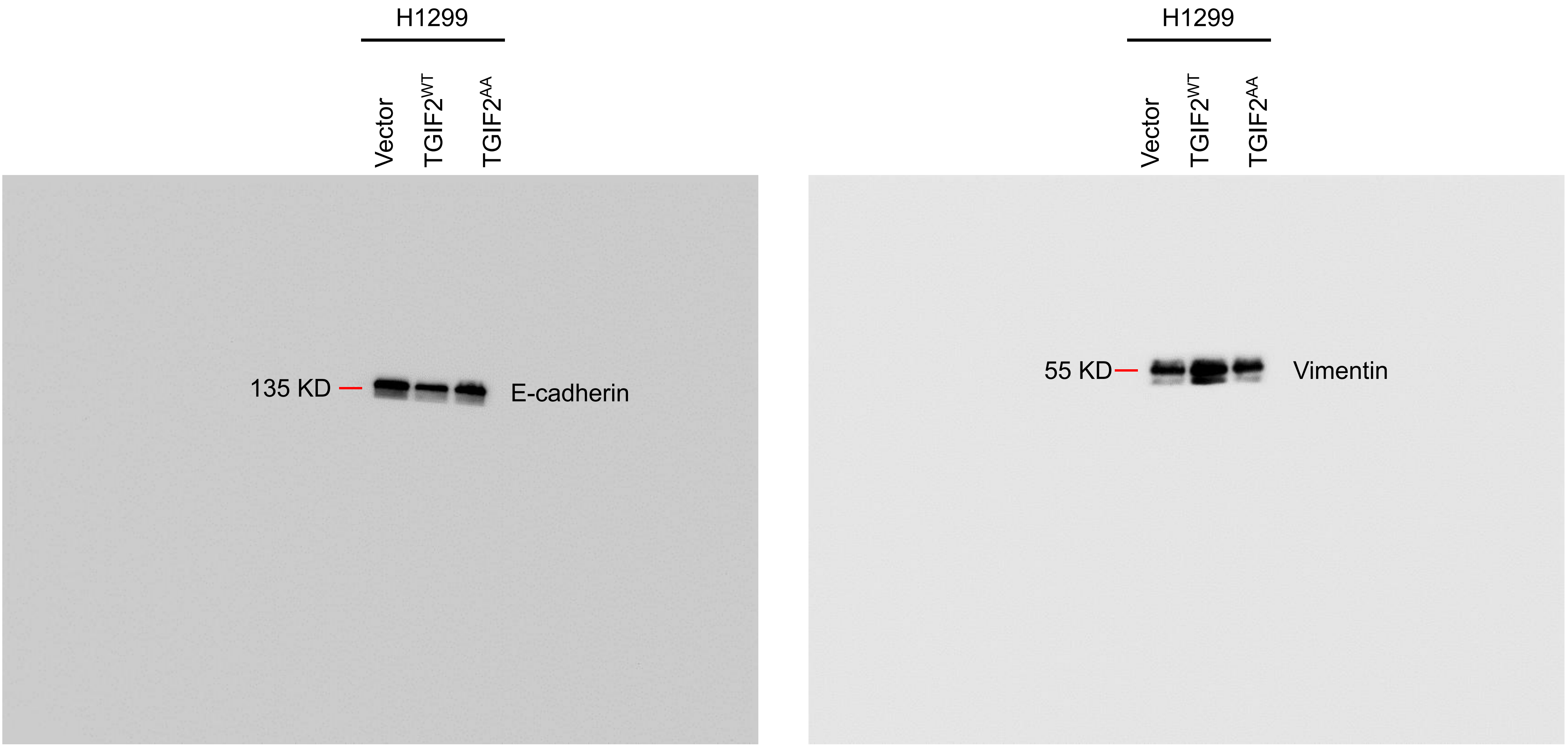

H1299
Vector
TGIF2WT
TGIF2AA
135 KD
E-cadherin
H1299
Vector
TGIF2WT
TGIF2AA
55 KD
Vimentin

Figure 3E

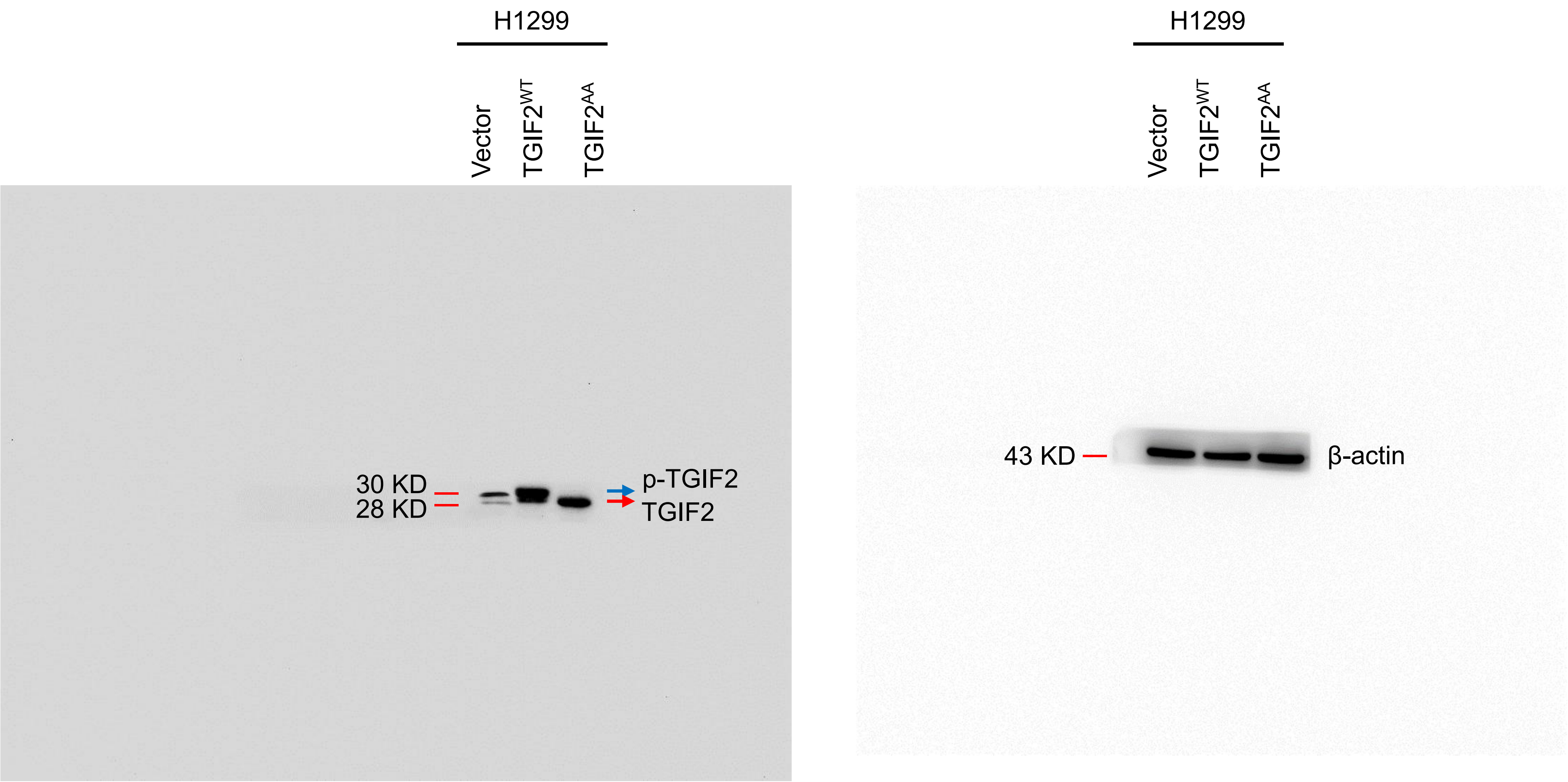

H1299
Vector
TGIF2^WT
TGIF2^AA
30 KD
28 KD
p-TGIF2
TGIF2
H1299
Vector
TGIF2^WT
TGIF2^AA
43 KD
β-actin

Figure 4G

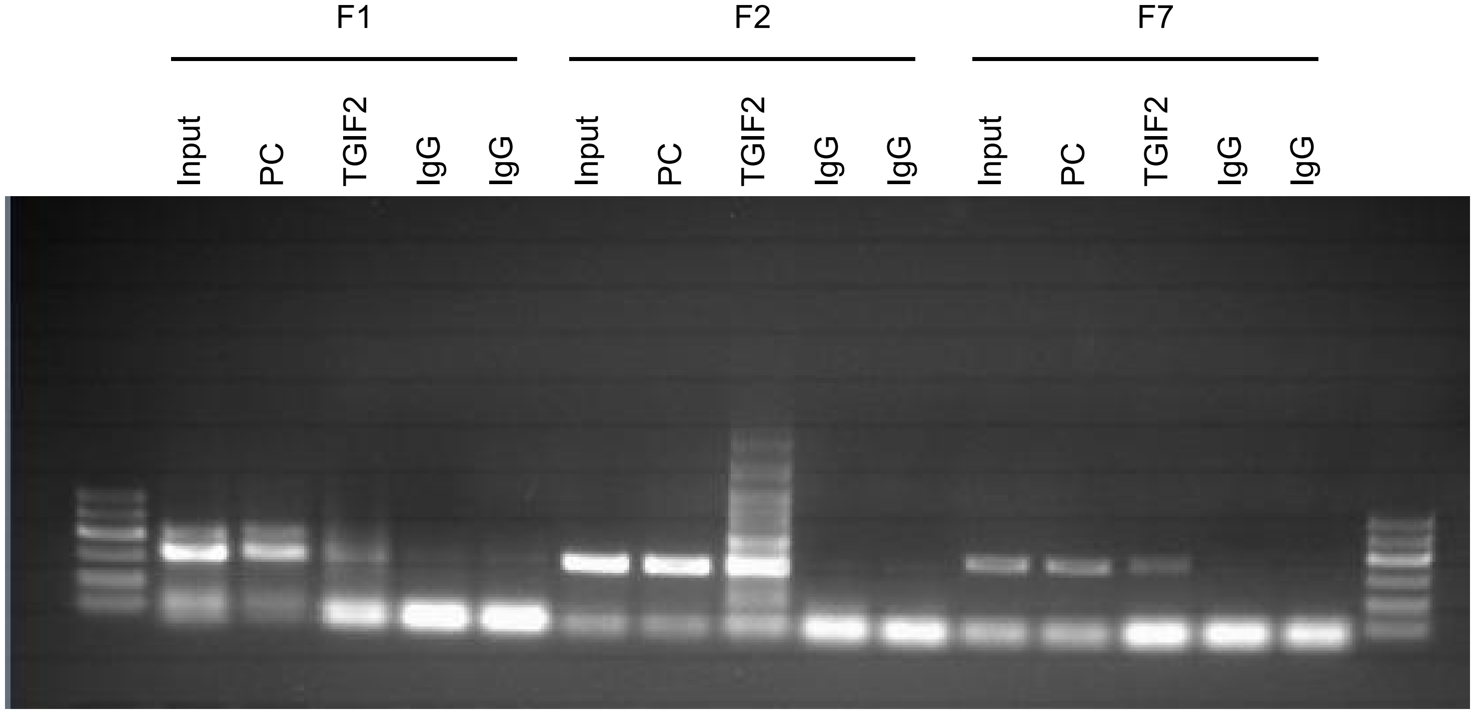

F1
F2
F7
Input
PC
TGIF2
IgG
IgG
Input
PC
TGIF2
IgG
IgG
Input
PC
TGIF2
IgG
IgG

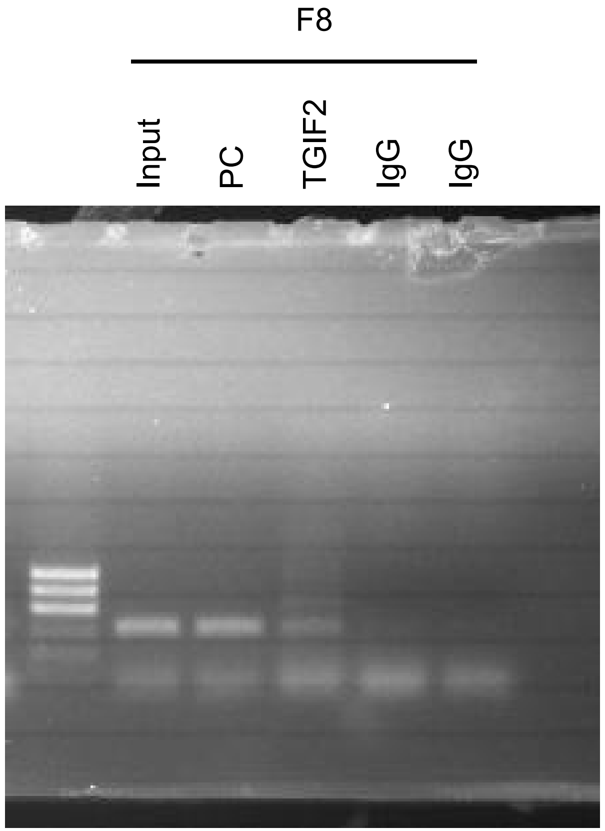

F8
Input
PC
TGIF2
IgG
IgG

Figure 5C

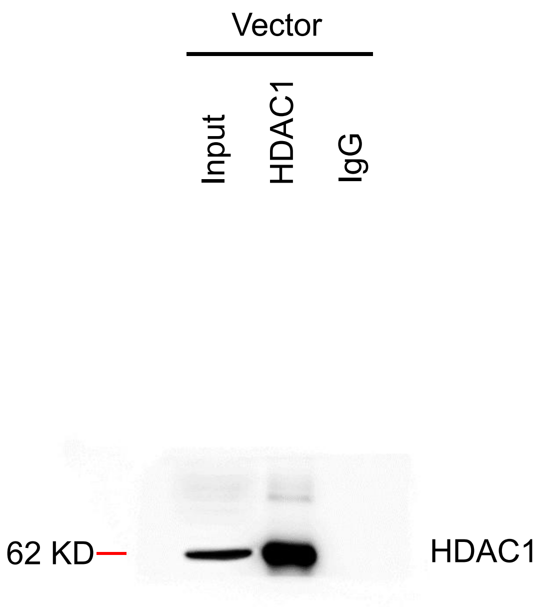
Vector
Input
HDAC1
IgG
62 KD
HDAC1

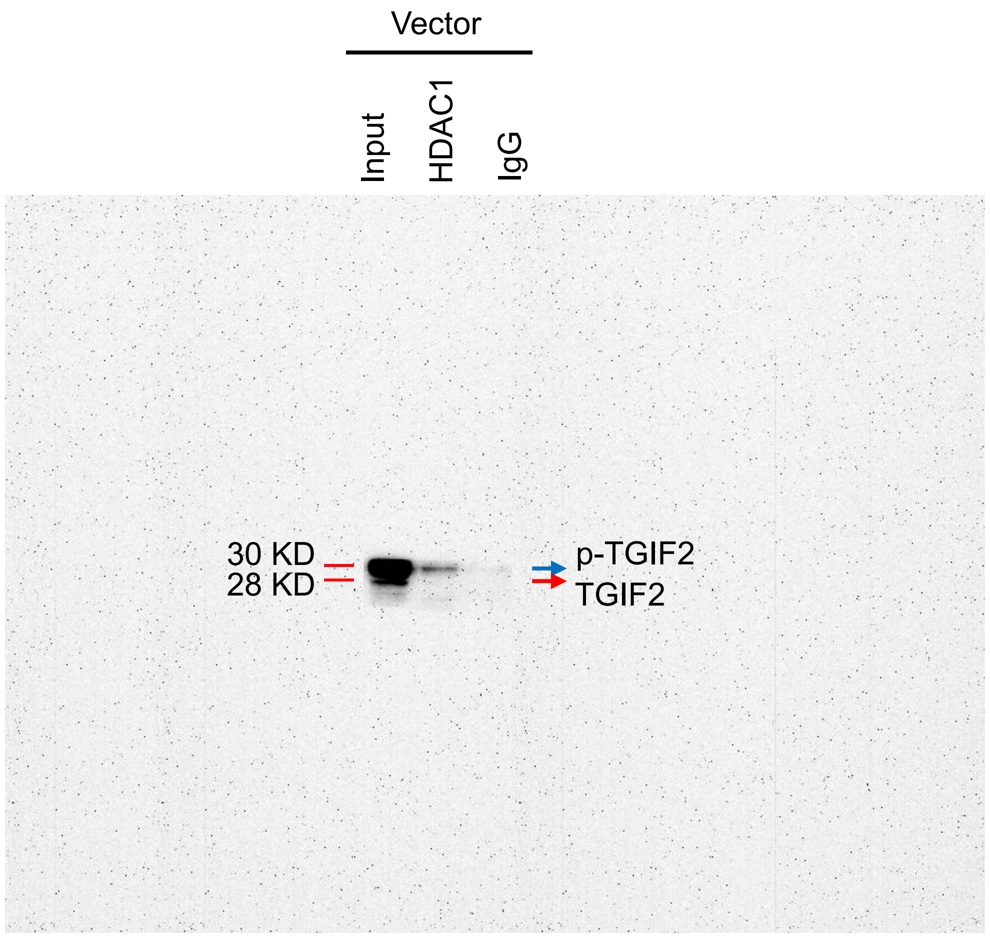
Vector
Input
HDAC1
IgG
30 KD
28 KD
p-TGIF2
TGIF2

Figure 5C

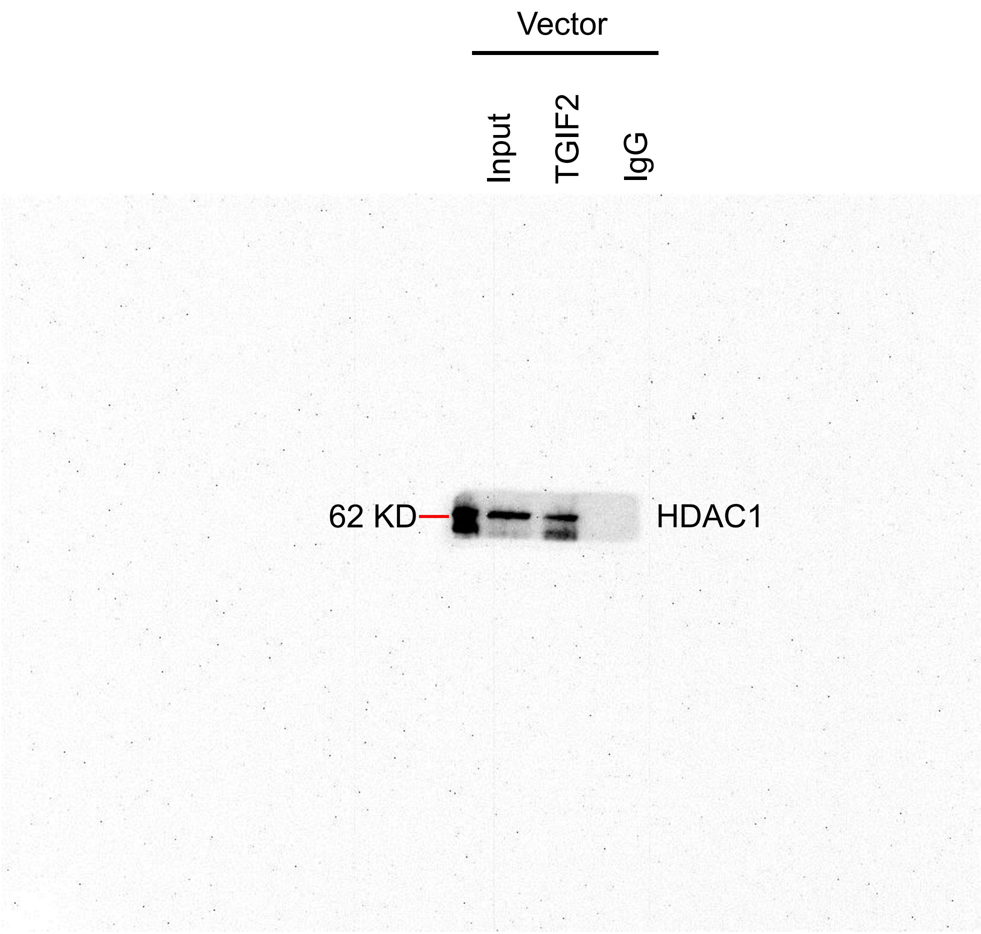

Vector
Input
TGIF2
IgG
62 KD
HDAC1

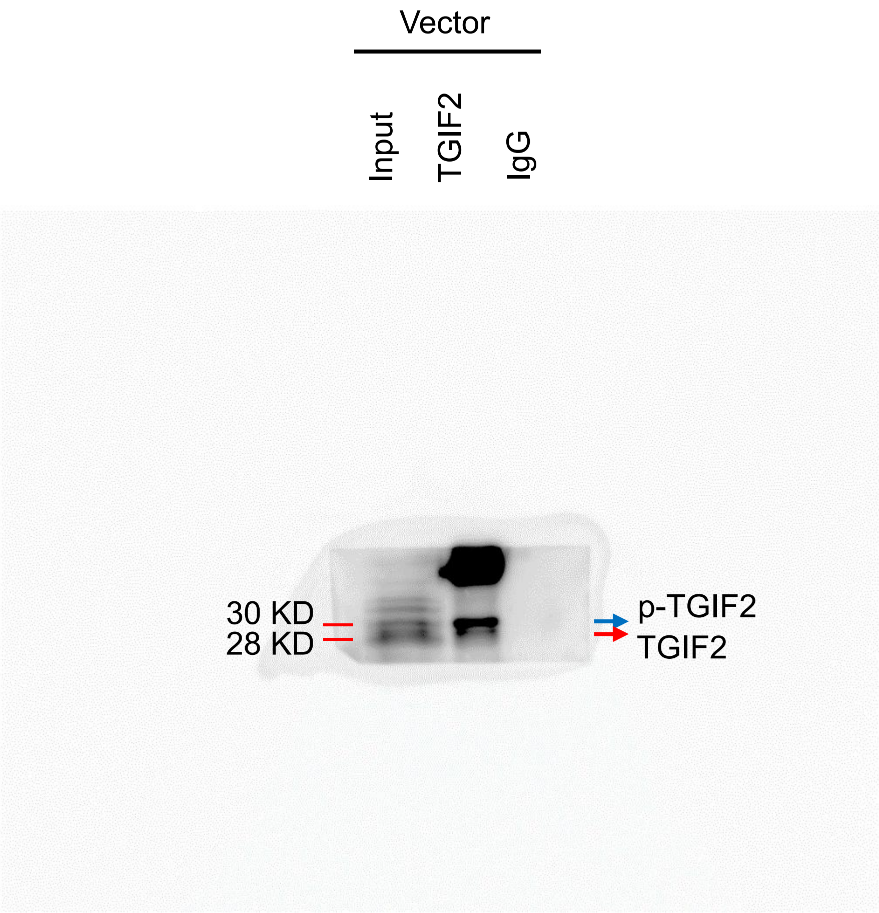

Vector
Input
TGIF2
IgG
30 KD
28 KD
p-TGIF2
TGIF2

Figure 5D

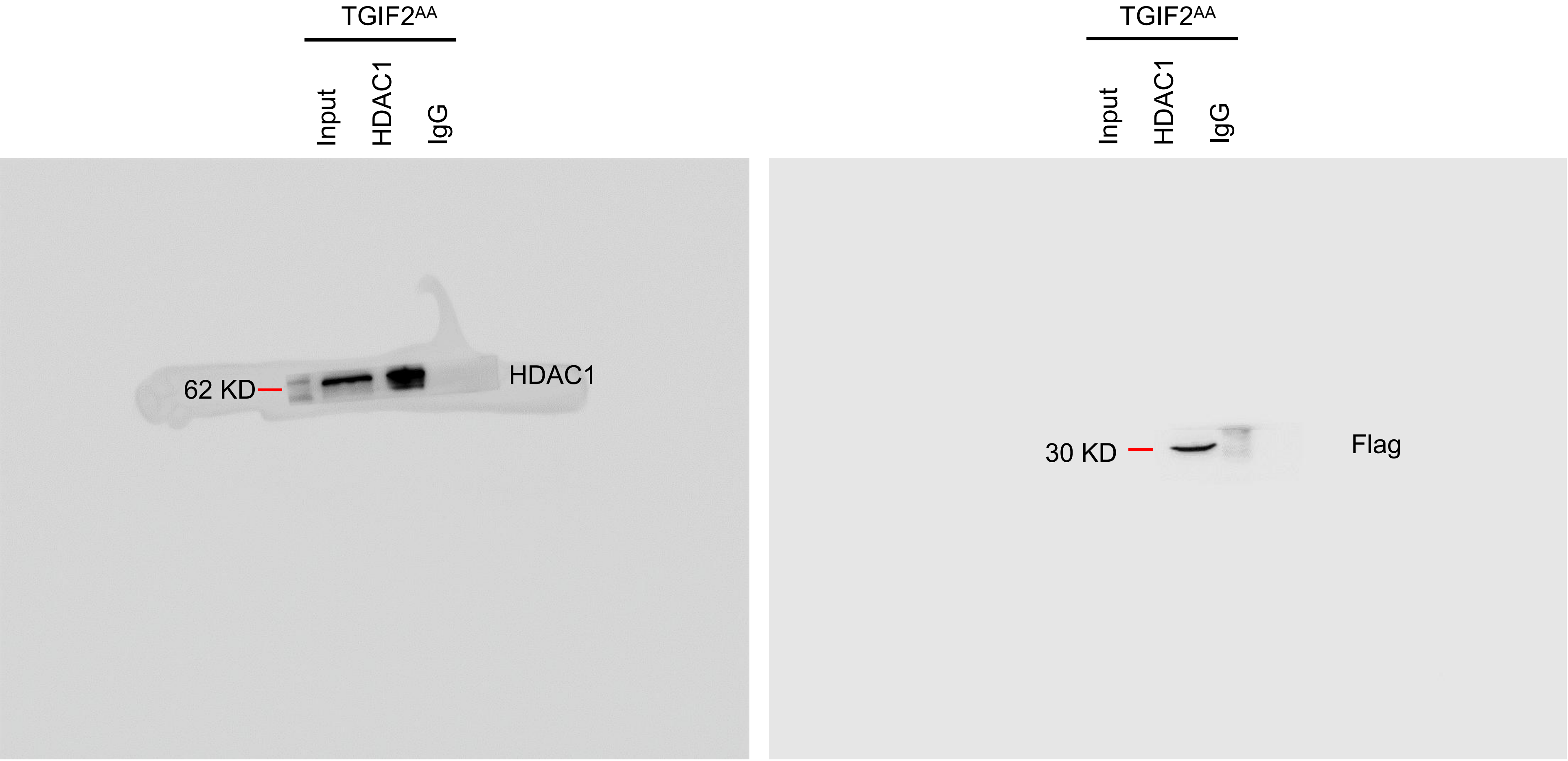
TGIF2AA
Input
HDAC1
IgG
62 KD
HDAC1
TGIF2AA
Input
HDAC1
IgG
30 KD
Flag

Figure 5D

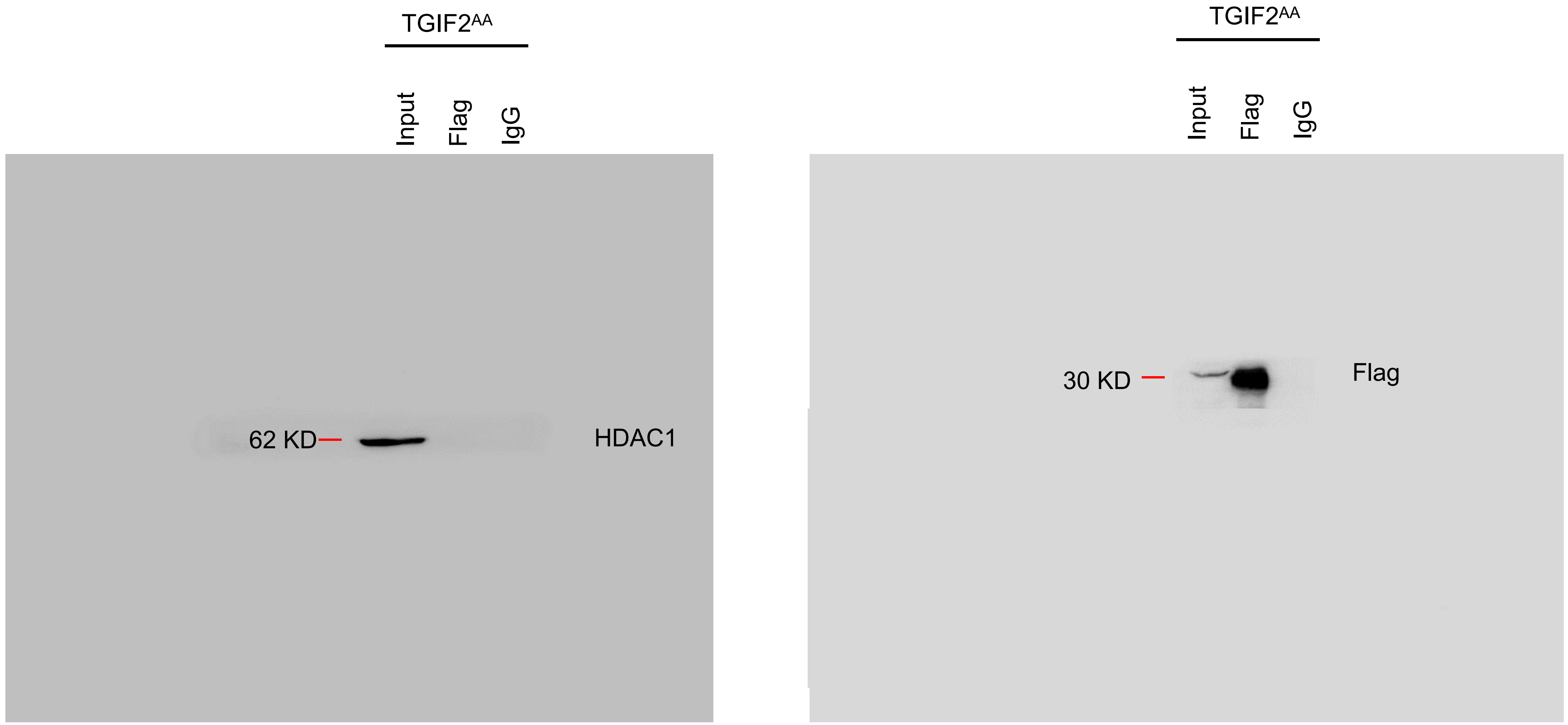
TGIF2AA
Input
Flag
IgG
62 KD
HDAC1
TGIF2AA
Input
Flag
IgG
30 KD
Flag

Figure 5F

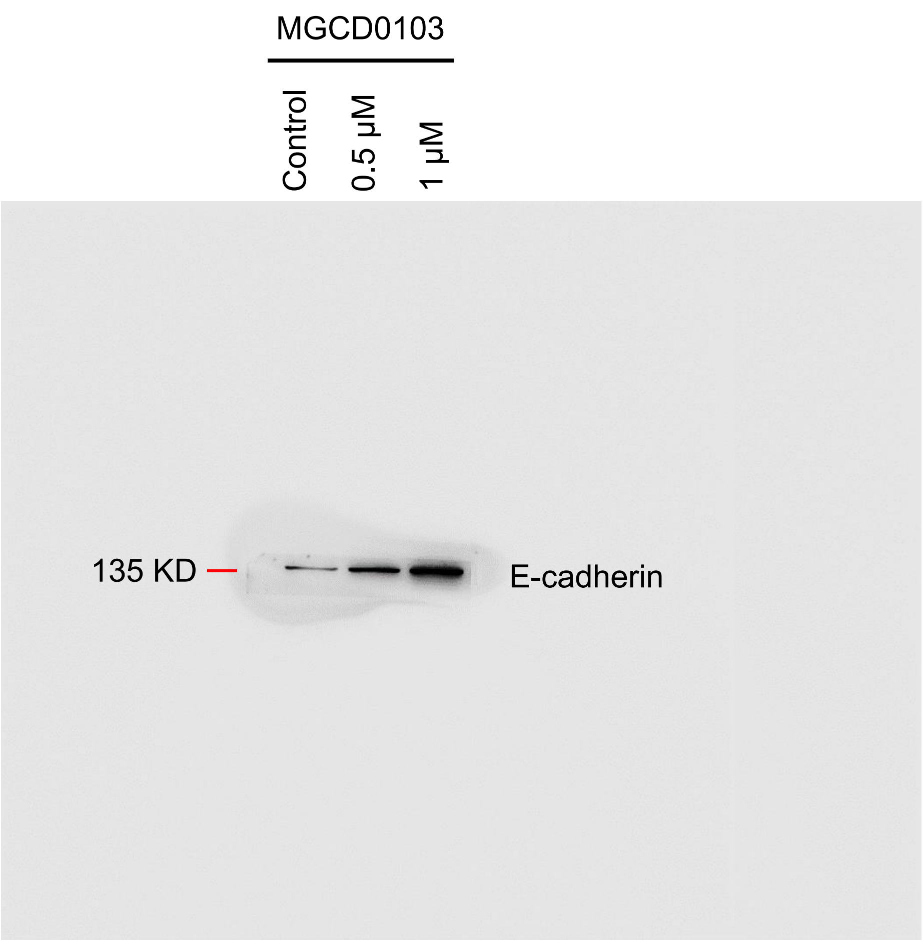

MGCD0103
Control
0.5 μM
1 μM
135 KD
E-cadherin

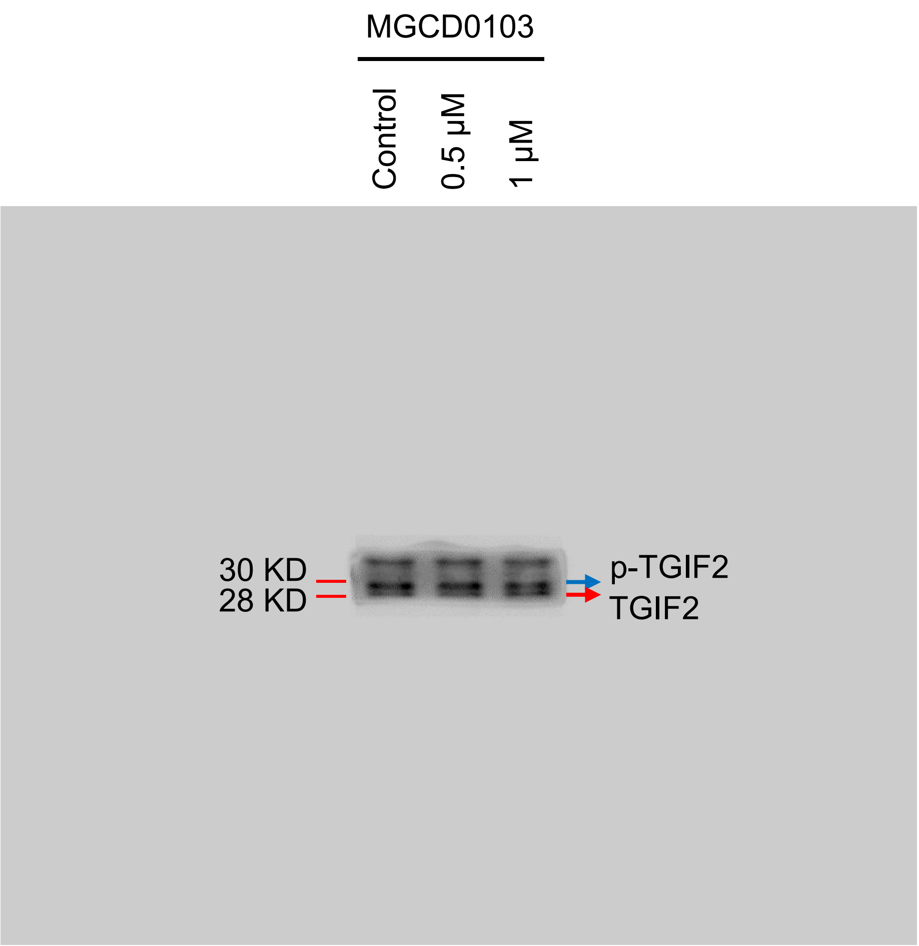

MGCD0103
Control
0.5 μM
1 μM
30 KD
28 KD
p-TGIF2
TGIF2

Figure 5F

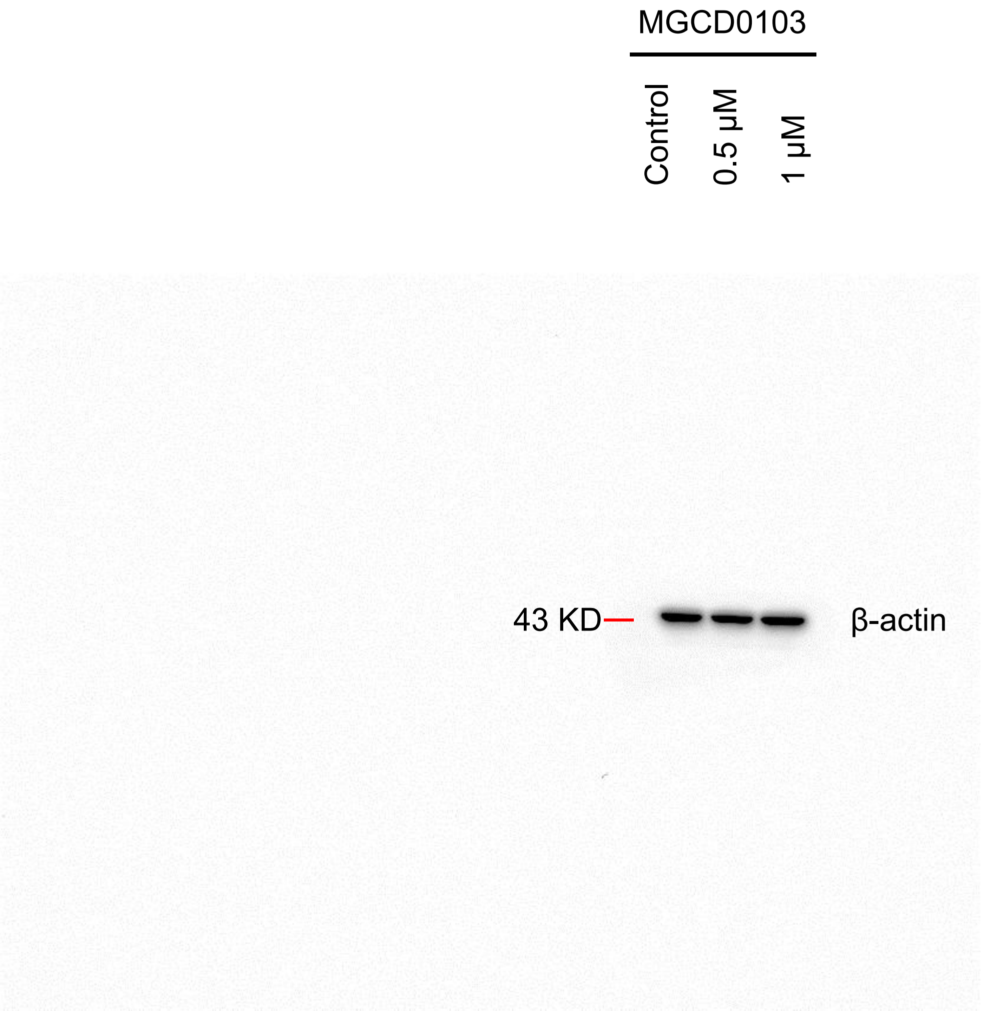

Figure 5G

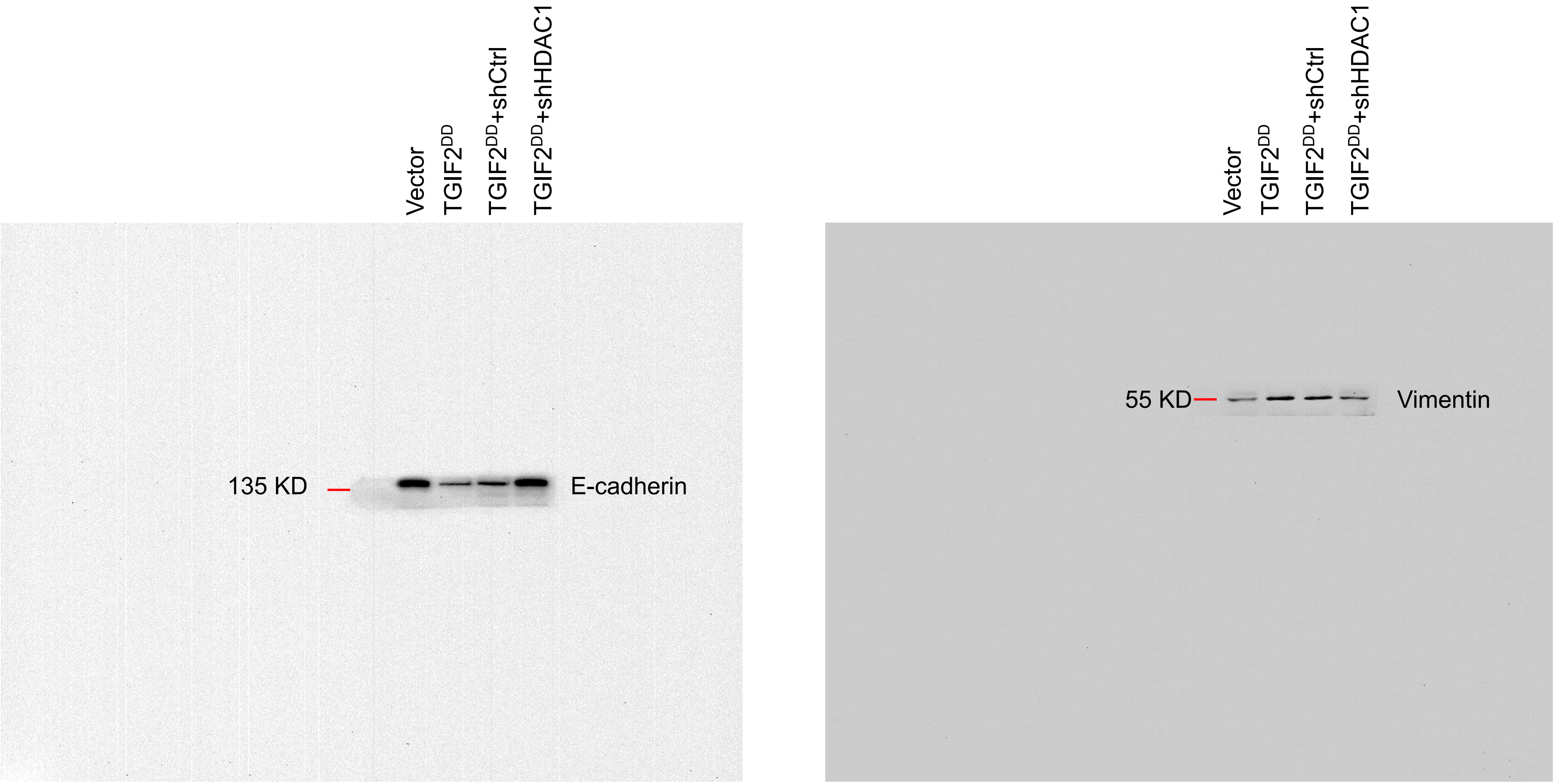
Vector
TGIF2DD
TGIF2DD+shCtrl
TGIF2DD+shHDAC1
135 KD
E-cadherin
Vector
TGIF2DD
TGIF2DD+shCtrl
TGIF2DD+shHDAC1
55 KD
Vimentin

Figure 5G

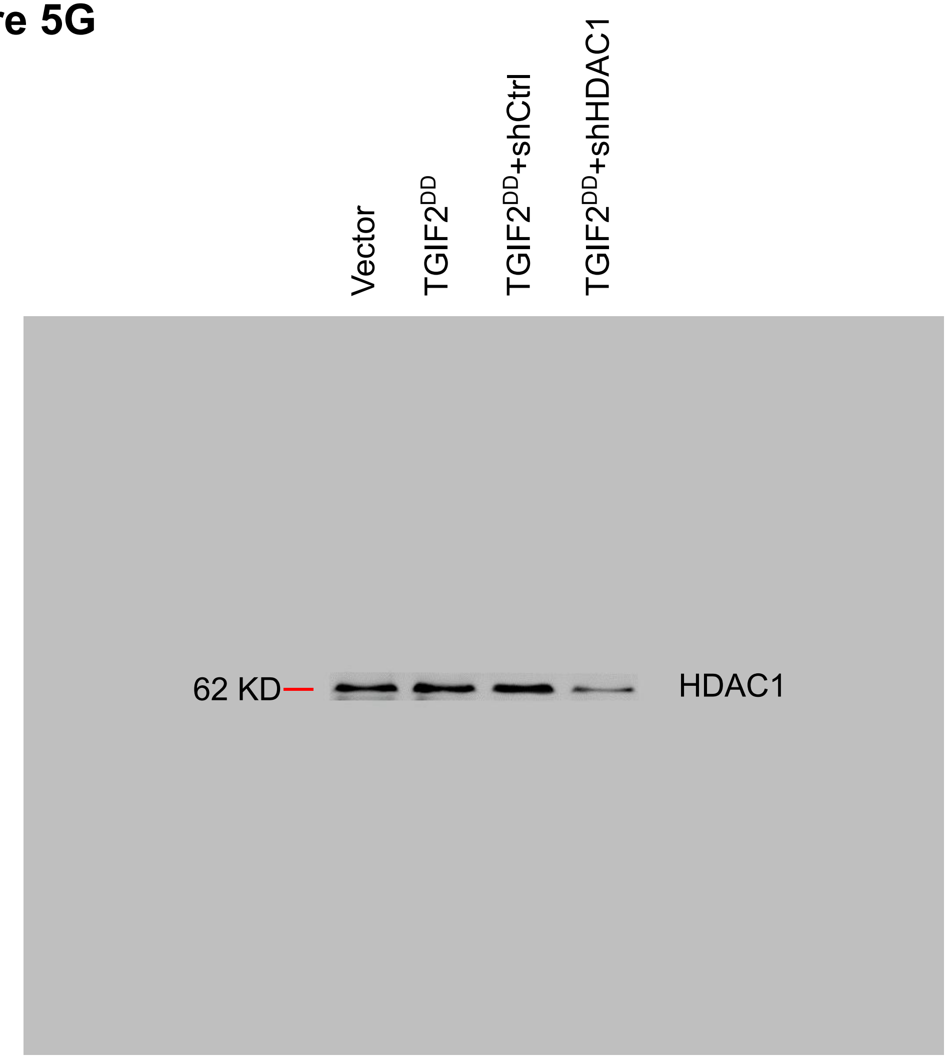

Vector
TGIF2DD
TGIF2DD+shCtrl
TGIF2DD+shHDAC1
62 KD
HDAC1

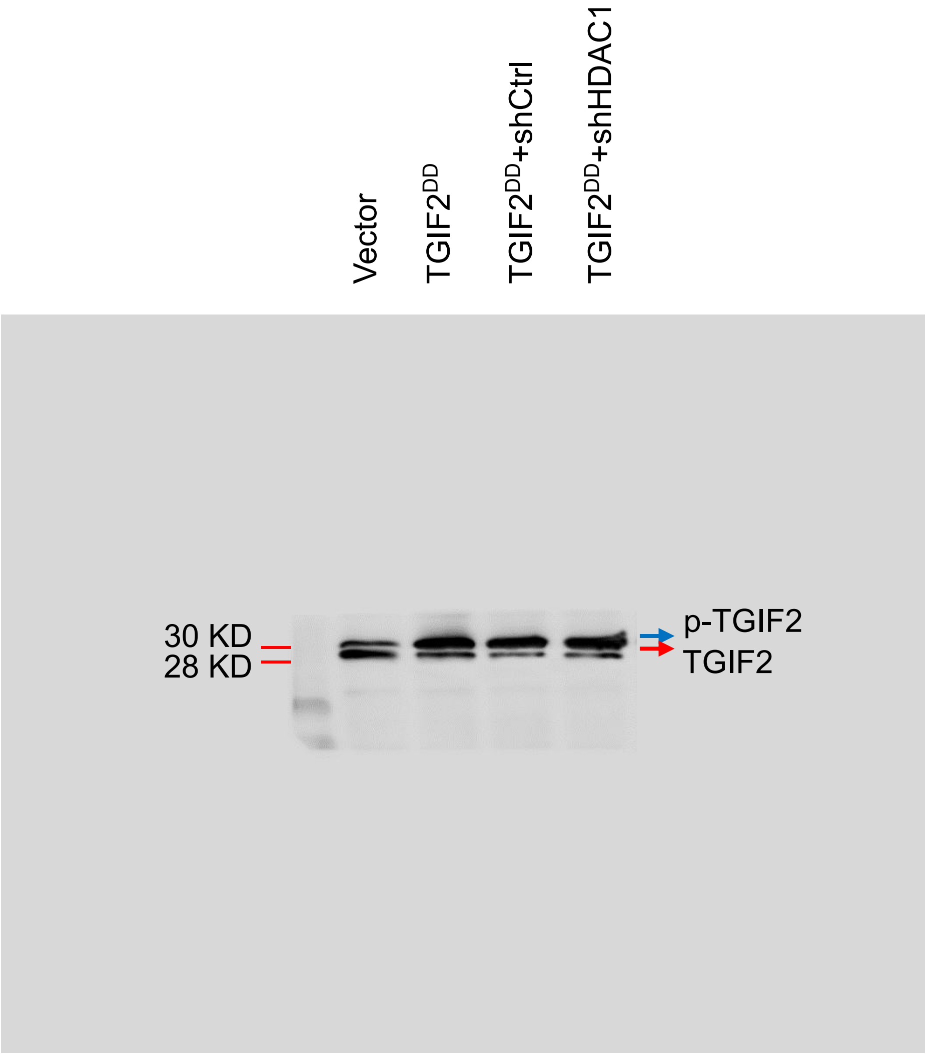

Vector
TGIF2DD
TGIF2DD+shCtrl
TGIF2DD+shHDAC1
30 KD
28 KD
p-TGIF2
TGIF2

Figure 5G

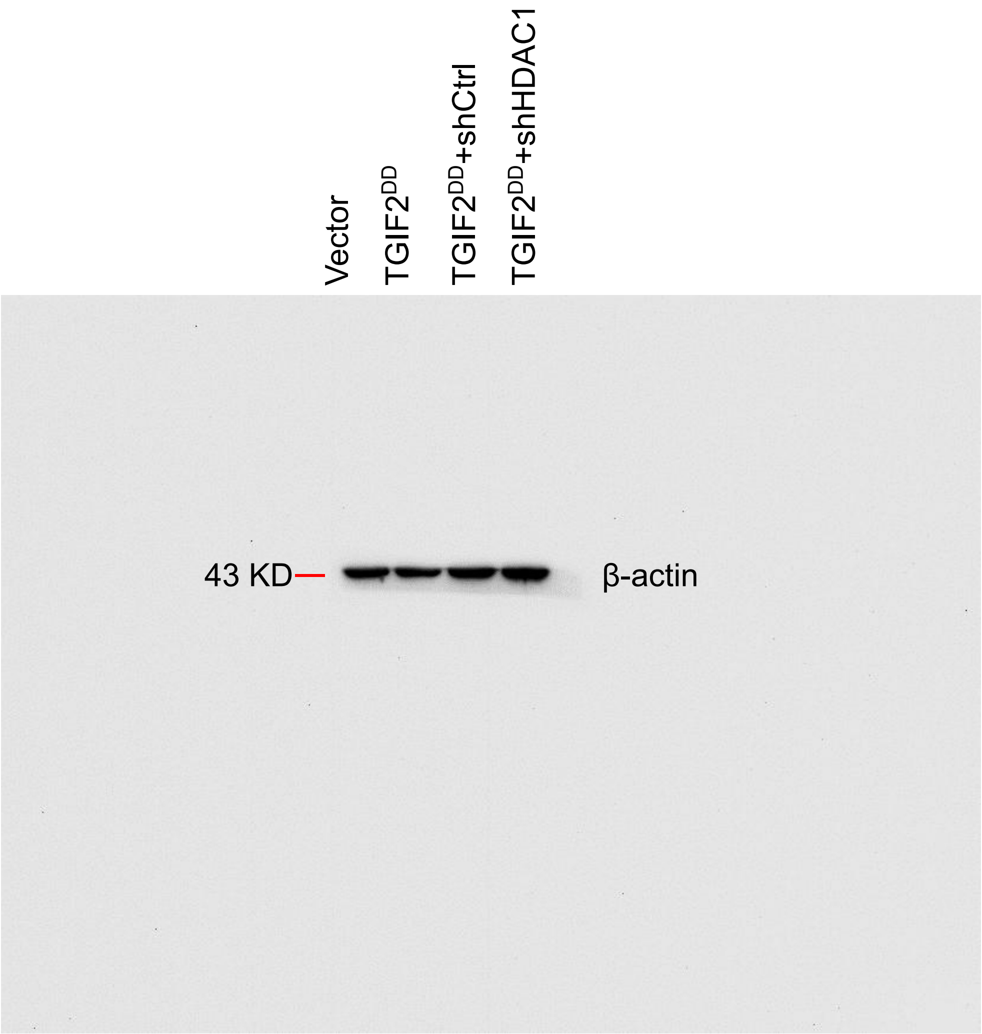

Vector
TGIF2DD
TGIF2DD+shCtrl
TGIF2DD+shHDAC1
43 KD
β-actin
