# Supplementary material for: Phosphorylation of TGIF2 represents a therapeutic target that drives EMT and metastasis of lung adenocarcinoma
Source: BMC Cancer. 2023 Jan 16;23:52. doi: 10.1186/s12885-023-10535-9 (PMC9841675; doi:10.1186/s12885-023-10535-9)
Supplement: Supplementary file 2 — Additional file 2. [file 12885_2023_10535_MOESM2_ESM.docx]

**­­­­­** Supplementary Materials for

(This file contains supplementary figures S1–S3, supplementary table S1–S4.)

Phosphorylation of TGIF2 represents a therapeutic target that drives EMT and metastasis of lung adenocarcinoma

Renle Du^1,2,4,5^, Chen Wang^3^, Jingjing Liu^1,5^, Keyan Wang^1,4^, Liping Dai^1,5*^ and Wenzhi Shen^6*^.

^1^Henan Institute of Medical and Pharmaceutical Sciences, Zhengzhou University, Zhengzhou 450052, China.

^2^College of Public Health, Zhengzhou University, Zhengzhou 450052, Henan, China.

^3^School of Basic Medical Sciences, Zhengzhou University, Zhengzhou 450052, China.

^4^State Key Laboratory of Esophageal Cancer Prevention & Treatment, Zhengzhou University, Zhengzhou 450052, Henan, China.

^5^Henan Key Medical Laboratory of Tumor Molecular Biomarkers, Zhengzhou University, Zhengzhou 450052, Henan, China.

^6^Department of Pathology and Institute of Precision Medicine, Jining Medical University, Jining 272067, China.

* Correspondence to: Wenzhi Shen, E-mail: shenwenzhi2011@126.com; Liping Dai, E-mail: lpdai@zzu.edu.cn.

**Figure S1. High TGIF2 expression was associated with poor clinical outcome in patients with LUAD.
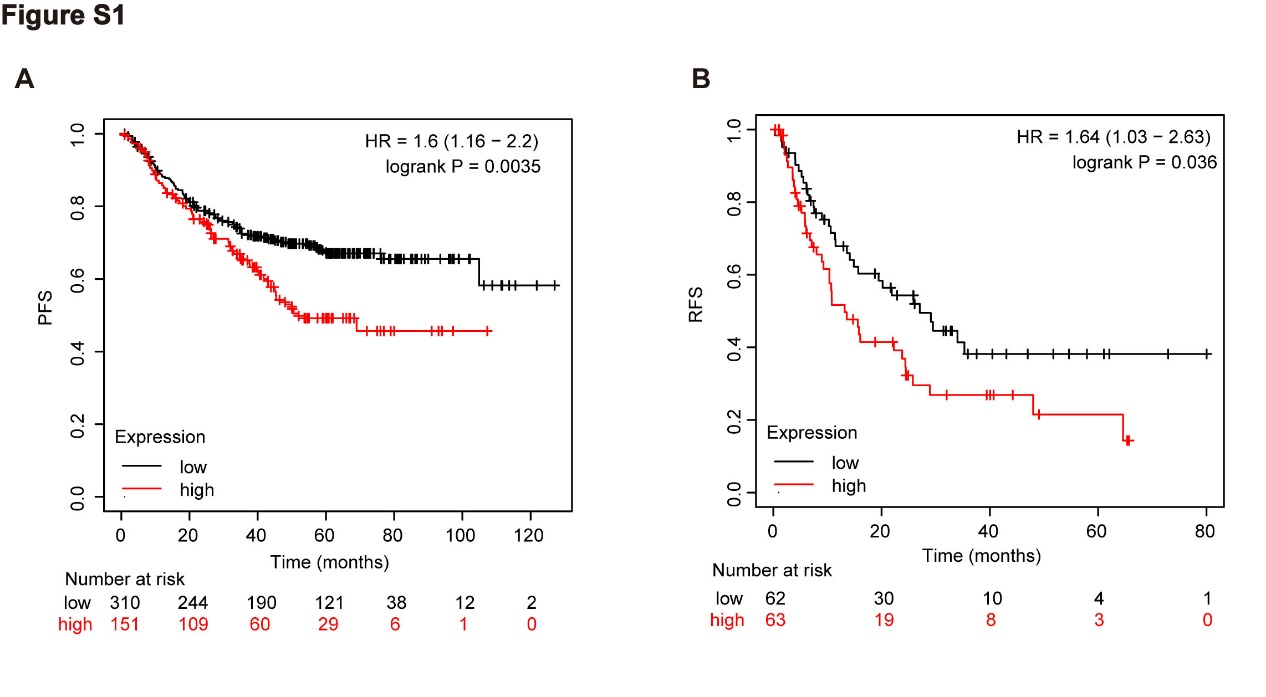
(A, B)** Kaplan–Meier curves showing the correlation between the level of TGIF2 and the first progression (FP) **(A)** and post progression survival (PPS) **(B)** of patients with lung adenocarcinoma ([log-rank test], HR, hazard ratio).


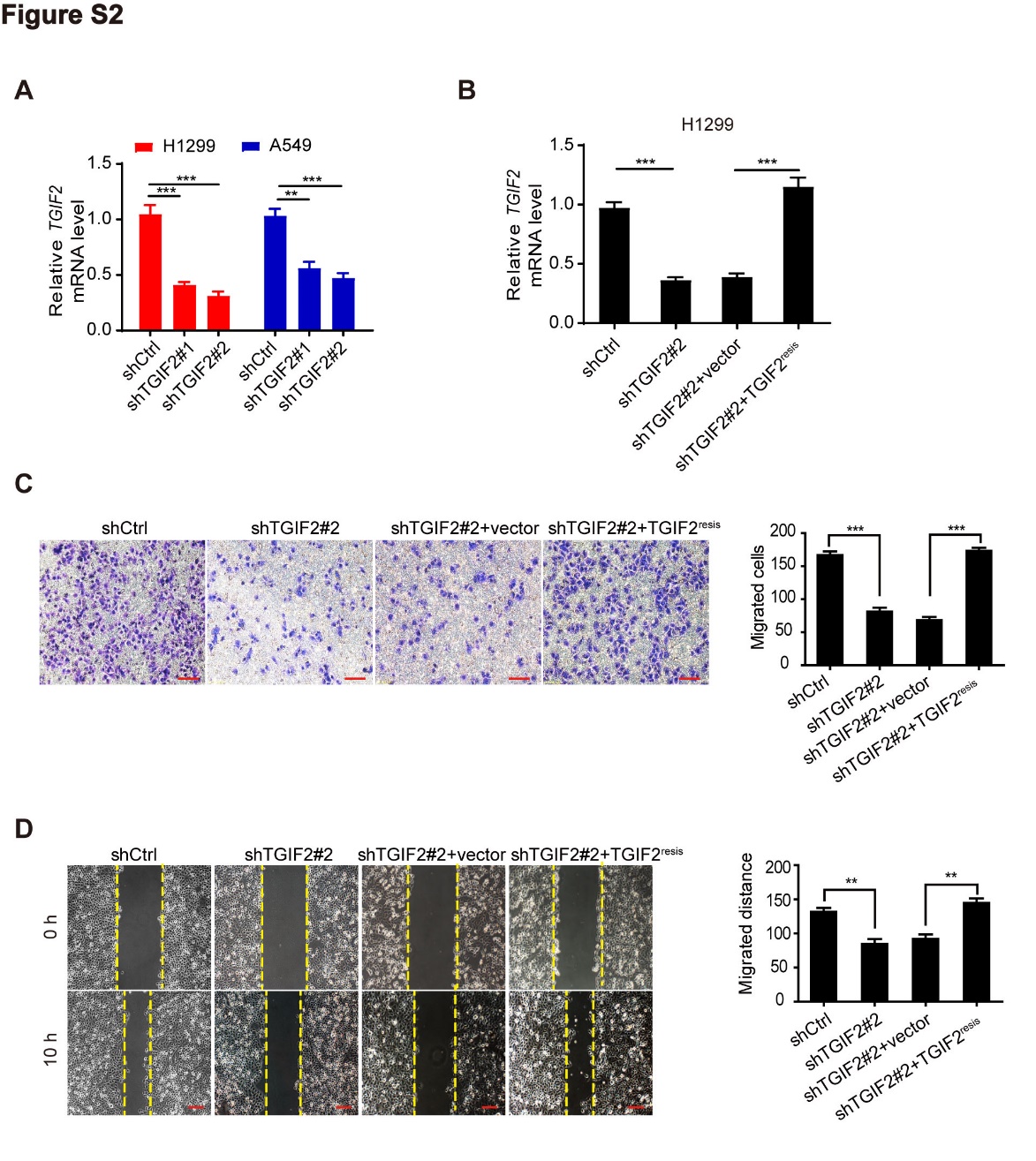


**Figure S2. TGIF2-knockdown decreased cell migrated ability is rescued by the ectopic expression of shRNA-resistant TGIF2.** (**A**) qRT-PCR analysis of *TGIF2* mRNA levels in H1299 and A549 cells stably transfected with two shRNAs targeting TGIF2. (**B**) qRT-PCR analysis of *TGIF2* mRNA levels in H1299 cells stably transfected with shTGIF2#2 and shTGIF2#2-resistant TGIF2 mRNA. (**C**) Transwell assay showing the migrated cells of the indicated stable H1299 cell strains. Scale bars, 100 μm. (**D**) Wound healing assay showing the migrated distance of the indicated stable H1299 cell strains. Scale bars, 100 μm. **, *p* < 0.01; ***, *p* < 0.001.


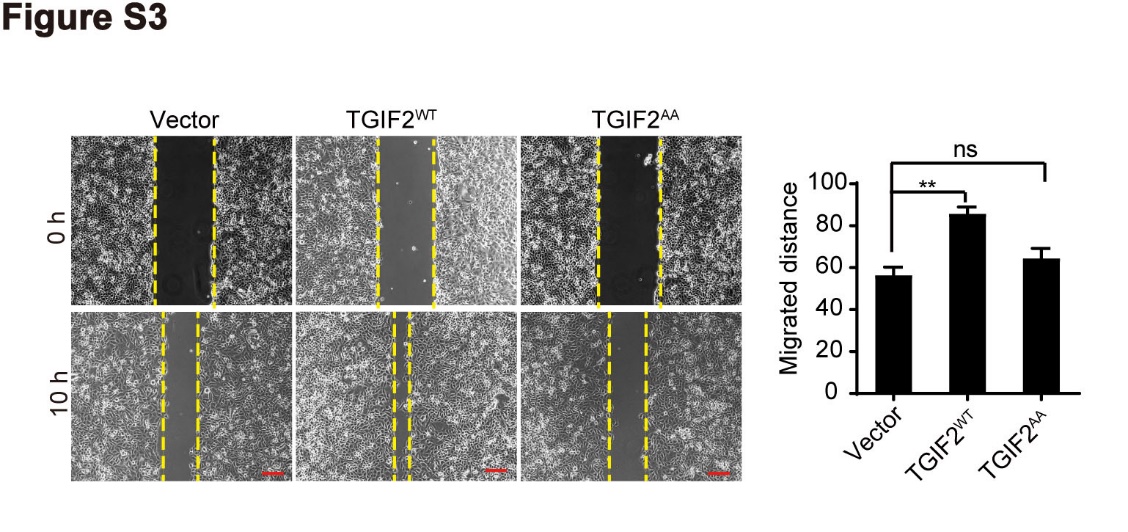


**Figure S3. Phosphorylation-deficient TGIF2 lose the ability to promote LUAD cell migration.** Wound healing assay showing the migrated distance of the indicated stable H1299 cell strains. Scale bars, 100 μm. **, *p* < 0.01; ns, not significant.

Supplementary table S1. Primer sequences for real-time PCR

| **Gene** | **Forward Sequence (5’-3’)** | **Reverse Sequence (5’-3’)** |
| --- | --- | --- |
| ***E-cadherin*** | CAGCACGTACACAGCCCTAA | TGCAACGTCGTTACGAGTCA |
| ***Vimentin*** | GGACCAGCTAACCAACGACA | AAGGTCAAGACGTGCCAGAG |
| ***Fibronectin*** | TCGTGCTTTGACCCCTACACG | CGGGAATCTTCTCTGTCAGCC |
| ***U-Plasminogen Activator*** | CCAAAATGCTGTGTGCTGCT | GCCAGGCCATTCTCTTCCTT |
| ***Slug*** | ACGCCTCCAAAAAGCCAAAC | ACTCACTCGCCCCAAAGATG |
| ***MMP9*** | TCTATGGTCCTCGCCCTGAA | CATCGTCCACCGGACTCAAA |
| ***Snail*** | AAGATGCACATCCGAAGCCA | CATTCGGGAGAAGGTCCGAG |
| ***ZEB1*** | GTGACGCAGTCTGGGTGTAA | AGTGGAGGAGGCTGAGTAGG |
| ***Twist*** | CTCGGACAAGCTGAGCAAGA | GCTCTGGAGGACCTGGTAGA |
| ***TGIF2*** | CGGACAGTGATCTAGGTGAGGACG | GGGAAATGGTAAACTGATTAGGGTCTT |

Supplementary table S2. Primer sequences for clone

| **Clone** | **Forward Sequence (5’-3’)** | **Reverse Sequence (5’-3’)** |
| --- | --- | --- |
| **Human *CDH1* promoter** | CTCATGGCTCACACCTGAAA | AGTACAGGTGCACACCACCA |

Supplementary table S3. Primer sequences for ChIP-qPCR

| **Fragments of *CDH1* promoter** | **Forward Sequences (5’- 3’)** | **Reverse Sequences (5’- 3’)** |
| --- | --- | --- |
| **–6kb~-5kb** | GGAGGCGGTATAGCCAGTTC | GCTCCTGGCTAGGTAGGGTA |
| **–5kb~-4kb** | CCACTGTTAGCGCTGGATCT | TGTCCGTGCAGGTGATAAGG |
| **–4kb~-3kb** | GGGCCCAGCGATGGTATAAA | AGGGAGAAGGCTCCGGTATT |
| **–3kb~-2kb** | GGTAGACAAGCCACCCAGAC | CGCCGTCCTCCATCAAAAGA |
| **–2kb~-1kb** | CCACAACAGCATAGGGAGACA | CTAGGTCAGGACCACCTCCC |
| **–1kb~+1** | GAACCGTGCAGGTCCCATAA | ATTGGCTGAGGGTTCACCTG |
| **+1~+1kb** | GCTCTGAGGAGTGGTGCATT | CAAGACCTAGCCCACCGTTC |
| **+1kb ~+2kb** | GGTAACCCTGCCTGGTTGTT | GTCTCTACAGTGCTGAGGGC |
| **+2kb ~+3kb** | TCCACTTGACTGTTGTCCAGG | ACACAGTGGAGGTGTTCCCT |

Supplementary table S4. Primer sequences for ChIP-PCR

| **Fragments of *CDH1* promoter** | **Forward Sequences (5’- 3’)** | **Reverse Sequences (5’- 3’)** |
| --- | --- | --- |
| **–6kb~-5kb** | AGAGGTTGCAGTGAGCCGAG | GCTGAGAAGAGTCCAACCGC |
| **–5kb~-4kb** | AGAGGTCTGGAGAGGGGCAC | TTTGGGAGGCTAGTTGGGAG |
| **–4kb~-3kb** | GTGCTGCGATTACAGTTGTGAG | TGTTTGGAATATTTCACCCAGAG |
| **–3kb~-2kb** | TGGCTTTCCCCAGGAGG | CGCCACCACGACTGGC |
| **–2kb~-1kb** | CATGCCTGTAATCCCAGCTACT | CCGGGTTCAAGAGACTCTCCT |
| **–1kb~+1** | CAGGCGGAGGTTGCAGTGA | GAGAGGGGGTGCGTGGCT |
| **+1~+1kb** | AGTGGCGTCGGAACTGCAA | CTGGGGTGGGGGAAAGGTA |
| **+1kb ~+2kb** | GTGAGGGCGCGCTGC | AGGATTACAGGCACACGCC |
| **+2kb ~+3kb** | GTGTGCCTGTAATCCTAGCTACTC | GCTCTAGAGCACACAGAGCAAAT |
